# Peniginsengins B–E, New Farnesylcyclohexenones from the Deep Sea-Derived Fungus *Penicillium* sp. YPGA11

**DOI:** 10.3390/md16100358

**Published:** 2018-10-01

**Authors:** Zhongbin Cheng, Wei Xu, Lijun Liu, Shumin Li, Wangjun Yuan, Zhuhua Luo, Jingjie Zhang, Yongjun Cheng, Qin Li

**Affiliations:** 1 College of Pharmacy, Henan University, Kaifeng 475004, China; czb360@126.com (Z.C.); 15736871748@163.com (L.L.); lishumin417@163.com (S.L.); yuanwangjun@henu.edu.cn (W.Y.); zhang521571@163.com (J.Z.); chyj15926@163.com (Y.C.); 2 Key Laboratory of Marine Biogenetic Resources, Third Institute of Oceanography, State Oceanic Administration, Xiamen 361005, China; xuwei@tio.org.cn (W.X.); luozhuhua@tio.org.cn (Z.L.); 3 Eucommia Ulmoides Cultivation and Utilization of Henan Engineering Laboratory, Kaifeng 475004, China; 4 College of Chemistry and Chemical Engineering, Henan University, Kaifeng 475004, China

**Keywords:** *Penicillium* sp., deep sea-derived fungus, farnesylcyclohexenones, antibacterial

## Abstract

Chemical examination of the EtOAc extract of the deep sea-derived fungus *Penicillium* sp*.* YPGA11 resulted in the isolation of four new farnesylcyclohexenones, peniginsengins B–E (**1**–**4**), and a known analog peniginsengin A (**5**). The structures of compounds **1**–**4** were determined on the basis of comprehensive analyses of the nuclear magnetic resonance (NMR) and mass spectroscopy (MS) data, and the absolute configurations of **1**, **2**, and **4** were determined by comparisons of experimental electronic circular dichroism (ECD) with calculated ECD spectra. Compounds **1**–**5**, characterized by a highly oxygenated 1-methylcyclohexene unit and a (4*E*,8*E*)-4,8-dimethyldeca-4,8-dienoic acid side chain, are rarely found in nature. Compounds **2**–**4** exhibited antibacterial activity against *Staphylococcus aureus*.

## 1. Introduction

The genus *Penicillium* is widely distributed in all environments, and has become one of the most well-known genera of fungi for the discovery of bioactive compounds [1]. To date, *Penicillium* species have provided us with several major clinical drugs, such as the well-known antibacterial penicillin and the remarkable lipid-lowering agent mevastatin. In recent years, marine-derived *Penicillium* have drawn attention of natural prodcuct chemists and have been increasingly investigated, leading to the discovery of thousands of secondary metabolites, some of which are structurally unique or biologically significant [2,3,4,5,6,7,8,9]. For example, simpterpenoid A, an unprecedented meroterpenoid isolated from mangrove-derived *Penicillium simplicissimum*, exhibited inhibitory activity against influenza neuraminidase with an IC_50_ value of 8.1 nM [4].

**Figure 1:**
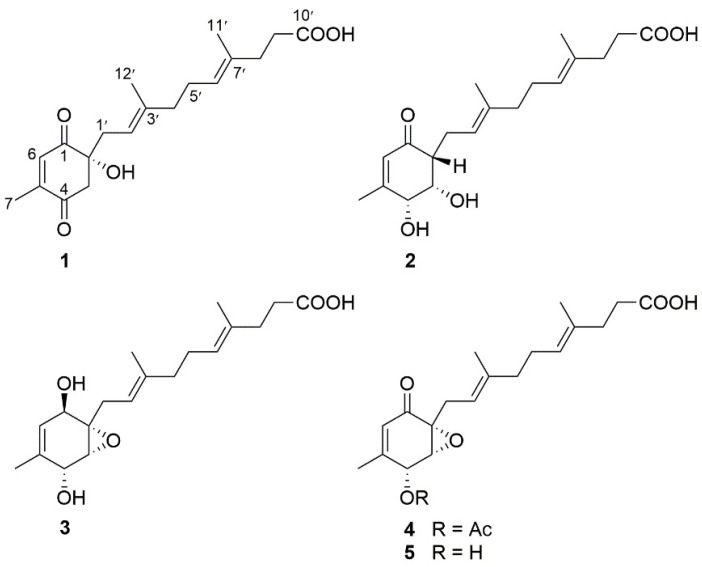
Structures of compounds **1**–**5** from *Penicillium* sp.

As part of our ongoing efforts to discover bioactive molecules from marine-derived fungi [10,11,12,13], a EtOAc extract of the fungal fermentation of the deep-sea-derived fungus *Penicillium* sp. YPGA11 possessed inhibitory effects against *Staphylococcus aureus* ATCC 25913 at a single dose (100 μg/mL). Further chromatographic separation of the extract yielded the known farnesylcyclohexenone peniginsengin A (**5**) [14], and four new analogs **1**–**4** (Figure 1). Compounds **1**–**4**, containing a highly oxygenated 1-methylcyclohexene unit and a rare (4*E*,8*E*)-4,8-dimethyldeca-4,8-dienoic acid side chain, are rarely found in nature [14,15]. The absolute configurations of this class of compounds were resolved for the first time by electronic circular dichroism (ECD) calculations in the current study. Compounds **1**–**4** were screened for their inhibitory effects against *Staphylococcus aureus* ATCC 25913 (methicillin-sensitive *S. aureus*, MSSA) and *Staphylococcus aureus* ATCC43300 (methicillin-resistant *S. aureus*, MRSA). Herein, the details of the isolation, structure elucidation, and antibacterial activity of **1**–**4** are described.

## 2. Results and Discussion

Peniginsengin B (**1**) had a molecular formula C_19_H_26_O_5_, as established by high-resolution electrospray ionization mass spectroscopy (HRESIMS) (333.1703 [M − H]**^−^**) and nuclear magnetic resonance (NMR) data (Supplementary Materials Figure S25 and Table 1), indicating seven degrees of unsaturation. The ^1^H NMR and heteronuclear single quantum coherence (HSQC) spectra provided the signals for three olefinic methyl groups (δ_H_ 1.89, 1.55, 1.48), three olefinic protons (δ_H_ 6.69, 5.02, 5.06), and a series of alkyl protons, while the ^13^C NMR spectrum exhibited a total of 19 carbon resonances, including six olefinic carbons for three double bonds (δ_C_ 135.7, 150.3, 118.4, 138.1, 123.9, and 133.7), three carbonyl carbons for two ketones (δ_C_ 197.1 and 199.6) and a carboxylic group (δ_C_ 174.2), and an oxyquaternary carbon (δ_C_ 77.1). As six of the seven degrees of unsaturation were accounted for by three double bonds and three carbonyl carbons, the remaining degree of unsaturation required that **1** was monocyclic. 

**Figure 2:**
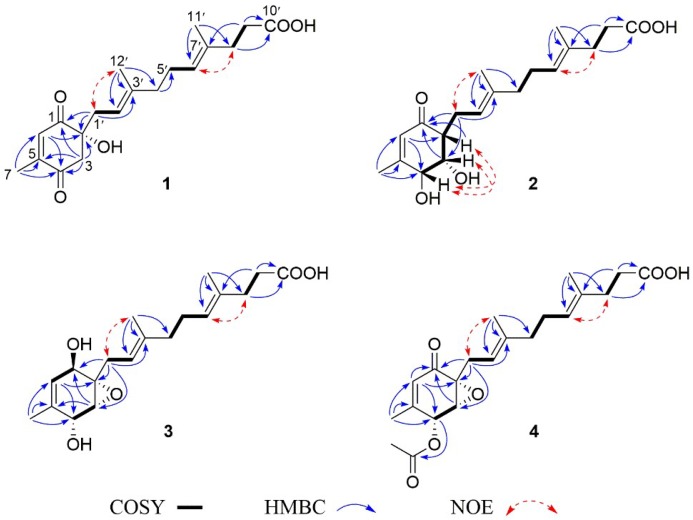
Key correlation spectroscopy (COSY), heteronuclear multiple bond correlation (HMBC), and nuclear Overhauser effect (NOE) correlations of **1**–**4**.

The aforementioned information was quite similar to that of the co-isolated compound, peniginsengin A (**5**) [14]. Comparison of their NMR data indicated that they shared the same side chain and the differences were attributed to the substitutions of the cyclohexenone moiety, which was established by heteronuclear multiple bond correlations (HMBC) (Figure 2). The HMBC correlations from the easily recognizable CH_3_-7 (δ_H_ 1.89) signal to C-4 (δ_C_ 197.1), C-5 (δ_C_ 150.3), and C-6 (δ_C_ 135.7) assigned an α,β-unsaturated ketone residing at C-4, C-5, and C-6 (Figure 2). Additional HMBC correlations from H-6 (δ_H_ 6.69) to the oxyquaternary carbon C-2 (δ_C_ 77.1) and C-4 and from the methylene protons H_2_-3 (δ_H_ 2.87, 2.80) to C-1 (δ_C_ 199.6), C-2, C-4, and C-5 led to the establishment of the cyclohexendione moiety. The structure of the side chain, containing two isoprene and one acetic acid fragments, was secured by analyses of correlation spectroscopy (COSY) and HMBC correlations (Figure 2). The side chain was connected to C-2 by HMBC correlations from H_2_-1′ (δ_H_ 2.35, 2.22) to C-1 and C-2. 

**Figure 3:**
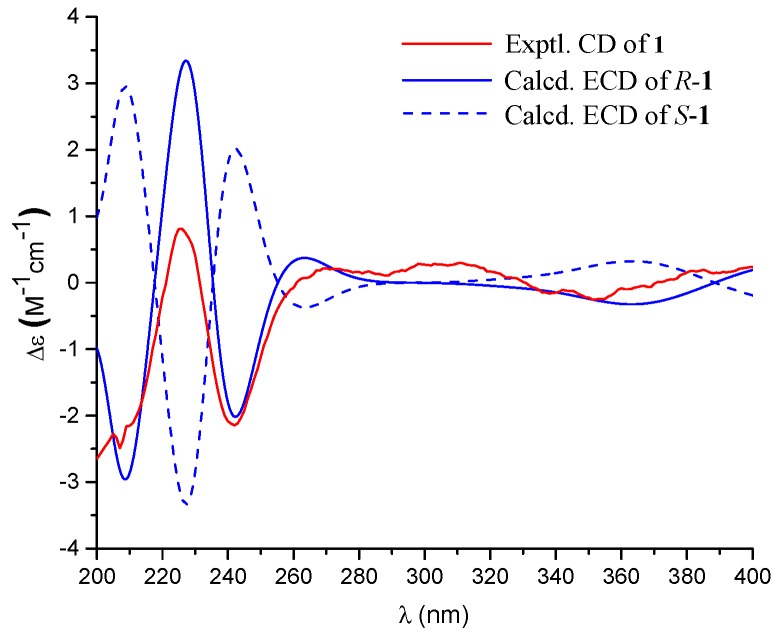
Experimental and calculated electronic circular dichroism (ECD) spectra of **1**.

The configurations of Δ^2^^′^ and Δ^6^^′^ were both assigned as *E* by the NOE correlations of H_2_-1′/H_3_-12′ (δ_H_ 1.48, s) and H-6′ (δ_H_ 5.06)/H_2_-8′ (δ_H_ 2.15) (Figure 2). The absolute configuration of the only chiral center of C-2 in **1** was resolved by comparing its experimental ECD spectrum with the calculated ECD spectra for the (2*S*/2*R*)-**1** using the time-dependent density functional theory (TDDFT) computational method (Figure 3). The experimental ECD spectrum of **1** showed an ECD curve with Cotton effects around 241 (−) and 226 (+) nm, respectively. The calculated ECD spectrum for (2*R*)-**1** showed a similar ECD curve with Cotton effects at 242 (−) and 227 (+) nm, which allowed the assignment of the absolute configuration of C-2 to be *R*. 

The molecular formula of **2** was determined as C_19_H_28_O_5_ by the HRESIMS data (*m*/*z* 335.1862 [M − H]**^−^**), indicating six degrees of unsaturation. The ^1^H NMR and ^13^C NMR data (Supplementary Materials Figure S26 and Table 1) showed signals for an α,β-methyl-unsaturated ketone (δ_H_ 5.84, 2.02; δ_C_ 201.4, 126.8, 162.4, 20.3), a methine (δ_H_ 2.43; δ_C_ 53.4), two oxymethines (δ_H_ 4.44, 4.20; δ_C_ 73.3, 73.7), and an identical side chain as that of **5.** These data showed high similarity to those of **5** with obvious differences owing to two downfield-shifted oxymethines (δ_C_ 73.3 and 73.7 in **2**; δ_C_ 68.0 and 59.4 in **5**) and the presence of one more methine (δ_C_ 53.4) in **2** instead of the oxyquaternary carbon (δ_C_ 61.9) in **5**, suggesting that **2** was the epoxy-hydrogenated derivative of **5**. The HMBC correlations from H-6 (δ_H_ 5.84) and H_3_-7 (δ_H_ 2.02) to C-4 (δ_C_ 73.3), H-6 to C-2 (δ_C_ 53.4), and COSY correlations from H-2 (δ_H_ 2.43) to H-4 (δ_H_ 4.44) confirmed the deduction and clearly clarified the structure of the cyclohexenone moiety (Figure 2). The side chain determined by 2D NMR data (Figure 2) was linked to the cyclohexenone moiety at C-2 by HMBC correlations from H_2_-1′ (δ_H_ 2.25, 2.60) to C-1 (δ_C_ 201.4) and C-3 (δ_C_ 73.7) combining with the COSY correlations of H-2 with H_2_-1′. Thus, the gross structure of **2** was deduced as shown in Figure 1. 

**Figure 4:**
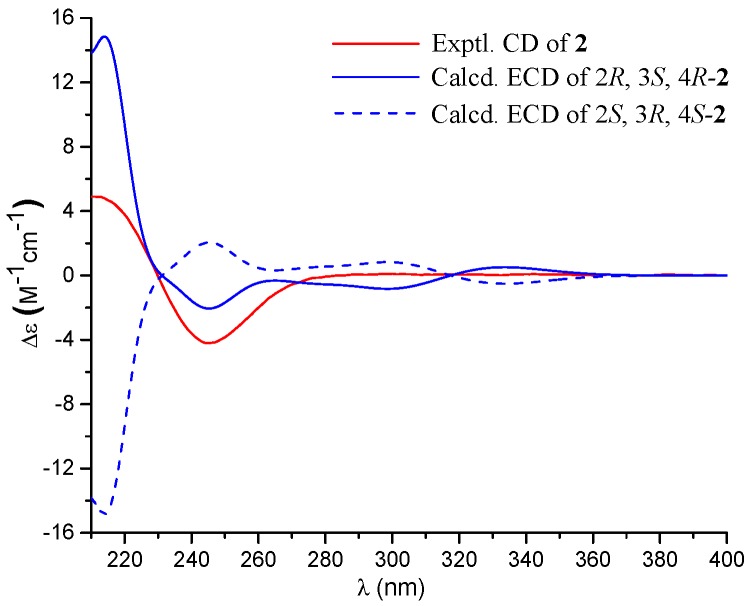
Experimental and calculated ECD spectra of **2**.

The relative configuration of **2** was determined by nuclear Overhauser effect spectroscopy (NOESY) data (Figure 2) and *J* values. The NOE correlations between H-2 and H-4 determined that they were in the same orientation. The coupling constant *J*_H-3/H-4_ (3.0 Hz) was indicative of the *cis*-relationship of H-3 and H-4 [6], which agreed with the NOE correlation between H-3 and H-4. Therefore, the relative configuration of **2** was assigned as 2*R*^∗^, 3*S*,^∗^ and 4*R*^∗^. Comparison of the experimental ECD spectra with those of the calculated ECD data for the model molecule with the 2*R*, 3*S*, and 4*R* configuration and its enantiomer allowed the assignment of the 2*R*, 3*S*, and 4*R* configurations for **2** (Figure 4).

Compound **3** had a molecular formula of C_19_H_28_O_5_ as determined by the HRESIMS (*m*/*z* 335.1868 [M − H]^−^) and NMR data (Supplementary Materials Figure S27 and Table 2). Comparison of the NMR data revealed the structure of **3** to be closely related to **5**. The difference was found by the presence of an additional oxymethine (δ_C_ 67.2) in **3** instead of the ketone group (δ_C_ 194.0) in **5**, indicating **3** was the carbonyl-hydrogenated derivative of **5**. This deduction was evident from the COSY relationship between H-1 (δ_H_ 4.18) and H-6 (δ_H_ 5.23) in addition to the HMBC correlations from H-3 (δ_H_ 3.31) and H-1′ (δ_H_ 2.90 and 2.12) to C-1 (δ_C_ 67.2) (Figure 2). The coupling constant *J*_H-3/H-4_ (2.3 Hz, in DMSO-*d*_6_, see Table S1) suggested a *cis* relationship between H-3 and H-4 and were assigned as α-oriented [16]. The OH-1 was determined to be β oriented by the absence of NOE correlation between H-3 and H-1 and was supported by comparison of the NMR data with those of craterellin D [17], a merosesquiterpenoid isolated from the soft coral-derived fungus *Lophiostoma* sp. Thus, the gross structure of **3** was established as shown in Figure 1. The absolute configuration of the stereogenic centers in **3** was proposed to be 1*R*, 2*S*, 3*R*, and 4*R* based on biogenetic consideration and by comparing its specific rotation with those of **1** and **2**. 

Compound **4** had a molecular formula of C_21_H_28_O_6_ as determined by the HRESIMS (*m*/*z* 375.1813 [M − H]^−^). A comparison of their NMR data revealed the structure of **4** closely related to **5** with the distinction by presence of an acetyl group (δ_H_ 2.20; δ_C_ 20.6, 171.9). The location of the acetyl group at C-4 was assigned by the HMBC correlation from H-4 (δ_H_ 5.85) to the acetyl carbonyl carbon (δ_C_ 171.9) in association with the COSY correlations between H-3 (δ_H_ 3.70) and H-4 (Figure 2). The similar NOE correlations and *J* values of **4** and **5** confirmed both compounds possessing the same relative configuration. Thus, **4** was the acetyl derivative of **5**.

**Figure 5:**
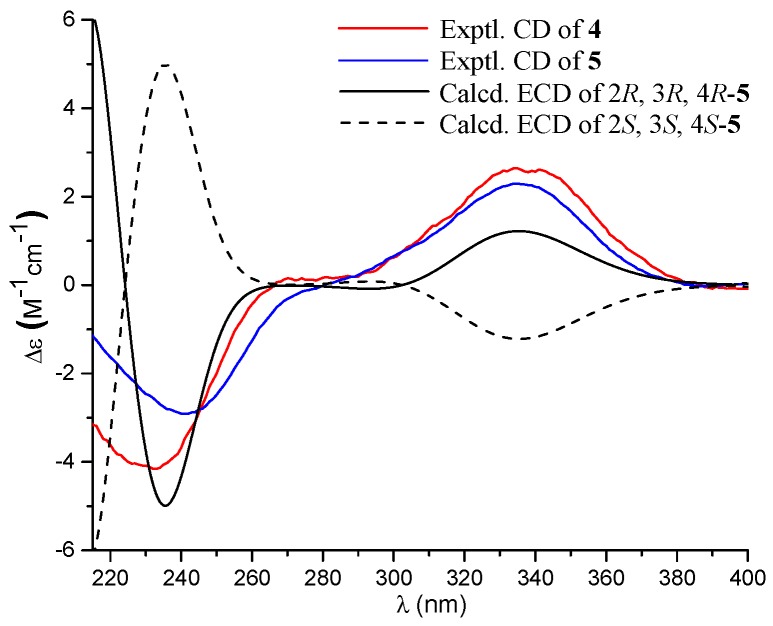
Experimental and calculated ECD spectra of **4** and **5**.

In the literature, the absolute configuration of **5** was left resolved. The ECD spectra of the model molecule with the 2*R*, 3*R*, and 4*R* configuration and its enantiomer were calculated (Figure 5), Comparison of the experimental ECD data of **5** with the calculated data for the model molecules indicated **5** to be in agreement with the 2*R*, 3*R*, and 4*R* configurations. In addition, the similar Cotton effects of **4** and **5** in the ECD spectra assigned **4** with the same absolute configuration as **5** (Figure 5).

Compounds **1**–**4** were tested for their antibacterial activity against *S*. *aureus* ATCC 25913 and *S. aureus* ATCC 43300. The results (Table 3) revealed that **2**–**4** possessed moderate activity against *S*. *aureus* ATCC 25913 with MIC values from 8 to 32 ug/mL. Compounds **2** and **4** exhibited inhibitory effect against *S*. *aureus* ATCC 43300 with MIC values of 32 and 64 ug/mL, respectively. 

Natural products featuring a highly oxygenated 1-methylcyclohexene unit connected with an isoprene-derived carboxylic acid side chain are rarely found in nature. Most cases belong to the well-known bioactive ambuic acid derivatives [18,19,20,21,22], which comprises the same conservative *E*-2-methylbut-2-enoic acid (5-carbon) side chain. The antibacterial polyketides penicyclones D and E, isolated from deep-sea-derived *Penicillium* sp., contain rare isoprene-derived 7-carbon side chains [6]. Peniginsengins A–E, bearing similar 1-methylcyclohexene moiety as those of ambuic acid derivatives and penicyclones, possess a distinctive (4*E*,8*E*)-4,8-dimethyldeca-4,8-dienoic acid (12-carbon) side chain and are rarely found in nature. Biogenetically, peniginsengins might derive from farnesylcyclohexene via oxidative cleavages in the farnesyl chain [23,24,25,26].

## 3. Experimental Section

### 3.1. General Experimental Procedure 

Specific rotations were measured by an SGW^®^-1 automatic polarimeter (Shanghai Jing Ke Industrial Co., Ltd., Shanghai, China). Ultraviolet (UV) spectra were measured on a UV-2600 spectrometer. ECD spectra were measured on an Aviv Model 420SF spectropolarimeter (Aviv Biomedical Inc., Lakewood, CO, USA). The NMR spectra were recorded on a Bruker Avance III HD-400 and HD 600 NMR spectrometers (Bruker, Fällanden, Switzerland). HRESIMS spectra were obtained on an AB Sciex Triple TOF 4600 spectrometer (AB Sciex Pte. Ltd., Redwood City, CA, USA) fitted with an electrospray ionization (ESI) source. Semi-preparative high-performance liquid chromatography (HPLC) was undertaken on a Shimadzu LC-6AD pump (Shimadzu Co., Kyoto, Japan) using a UV detector, and a YMC-Pack ODS-A HPLC column (semipreparative, 250 × 10 mm, S-5 μm, 12 nm, YMC Co., Ltd, Kyoto, Japan) was used for separation. 

### 3.2. Fungal Strain and Identification 

Fungus *Penicillium* sp*.* YPGA11 [13] (originally named YPCMAC1) was isolated from the deep-sea water at a depth of 4500 m in the Yap Trench (138° 0.74′ E, 8°0.36′ N, West Pacific Ocean). The strain was identified as *Penicillium* sp. based on microscopic examination and by internal transcribed spacer (ITS) sequencing. The ITS sequence has been deposited in GenBank (http://www.ncbi.nlm.nih.gov) with accession number MG835908. The strain YPE1 (MCCC 3A00982) was deposited at the Marine Culture Collection of China.

### 3.3. Fermentation 

The fermentation was carried out in 20 Fernbach flasks (500 mL), each containing 80 g of rice. Distilled H_2_O (100 mL) was added to each flask, and the contents were soaked overnight before autoclaving at 15 psi for 30 min. After cooling to room temperature, each flask was inoculated with 5.0 mL of the spore inoculum and incubated at 30 °C for 30 days.

### 3.4. Extraction and Isolation 

The fermented material was extracted with EtOAc (3 × 2000 mL). After evaporation under vacuum, the EtOAc extract (2.0 g) was subjected to ODS silica gel column chromatography (CC) eluting with MeOH/H_2_O (20:80→100:0) to obtain seven fractions (F1–F7). F5 (159 mg) was further chromatographed over C-18 silica gel CC eluted with MeOH/H_2_O (50:50→70:30) to to afford four subfractions (SF_a_–SF_d_). SF_a_ (41 mg) was purified by HPLC on a semipreparative YMC-pack ODS-A column using MeOH/H_2_O (65:35, 2 mL/min) as a mobile phase to obtain **3** (4.2 mg, t*_R_* 17.3 min) and **2** (2.1 mg, t*_R_* 18.2 min). SF_b_ (28 mg) was separated by HPLC using the YMC-pack ODS-A column eluted by MeOH/H_2_O (65:35, 2 mL/min) to afford **5** (3.0 mg, t*_R_* 39.5 min) and **1** (1.7 mg, t*_R_* 40.9 min). SF_d_ (31 mg) was subjected to HPLC on the YMC-pack ODS-A column with a mobile phase of MeCN/H_2_O (60:40, 2 mL/min) to yield **4** (1.6 mg, t*_R_* 28.5 min).

Peniginsengin B (**1**): colorless oil; [α]25D +136 (*c* 0.1, MeOH); UV (MeOH) *λ*_max_ 241 (3.97) nm; ECD (*c* 6.0 × 10^−4^ M, MeOH) *λ*_max_ (Δε) 226 (+1.42), 241 (−2.50); negative ESIMS *m*/*z* 333.47 [M − H]^−^, 667.51 [2M − H]^−^; ^1^H and ^13^C NMR data, see Table 1; HRESIMS *m*/*z* 333.1703 [M − H]^−^ (calcd. for C_19_H_25_O_5_^−^, 333.1707).

Peniginsengin C (2): colorless oil; [α]25D +165 (*c* 0.1, MeOH); UV (MeOH) *λ*_max_ 232 (3.27) nm; (*c* 8.9 × 10^−4^ M, MeOH) *λ*_max_ (Δε) 210 (+5.25), 245 (−4.37); ^1^H and ^13^C NMR data, see Table 1; negative ESIMS *m*/*z*; negative ESIMS *m*/*z* 335.44 [M − H]^−^, 671.51 [2M − H]^−^; HRESIMS *m*/*z* 335.1862 [M − H]^−^ (calcd. for C_19_H_27_O_5_^−^, 335.1864).

Peniginsengin D (3): colorless oil; [α]25D +88 (*c* 0.1, MeOH); ^1^H and ^13^C NMR data, see Table 2; negative ESIMS *m*/*z* 335.71 [M − H]^−^, 671.53 [2M − H]^−^; HRESIMS *m*/*z* 335.1868 [M − H]^−^ (calcd. for C_19_H_27_O_5_^−^, 335.1864).

Peniginsengin E (4): colorless oil; [α]25D +119 (*c* 0.1, MeOH); UV (MeOH) *λ*_max_ 240 (3.27) nm; ECD (*c* 4.0 × 10^−4^ M, MeOH) *λ*_max_ (Δε) 227 (−4.11), 331 (+2.90); positive ESIMS *m*/*z* 399.31 [M + Na]^+^; ^1^H and ^13^C NMR data, see Table 2; negative ESIMS *m*/*z* 751.58 [2M − H]^−^; HRESIMS *m*/*z* 375.1813 [M − H]^−^ (calcd. for C_21_H_27_O_6_^−^, 375.1813).

### 3.5. Computation Section

In general, conformational analyses were carried out via random searching in the Sybyl-X 2.0 using the MMFF94S force field with an energy cutoff of 2.5 kcal/mol. Subsequently, the conformers were re-optimized using density functional theory (DFT) at the B3LYP/6-31+G(d) level in MeOH using the polarizable conductor calculation model by the GAUSSIAN 09 program. The energies, oscillator strengths, and rotational strengths (velocity) of the first 30 electronic excitations were calculated using the TDDFT methodology at the B3LYP/6-311++G(d,p) level in MeOH. The ECD spectra were simulated by the overlapping Gaussian function (half the bandwidth at 1/e peak height, σ = 0.20, 0.20, 0.25 for **1**, **2**, and **5**, respectively). By comparing the experiment spectra with the calculated ECD spectra, the absolute configuration of **1**, **2**, **4**, and **5** was resolved.

### 3.6. Antibacterial Assay 

The strains used in antimicrobial tests were obtained from ATCC: *S. aureus* ATCC 43300 (methicillin-resistant *S. aureus*, MRSA) and *S. aureus* ATCC 29213 (methicillin-sensitive *S. aureus*, MSSA). The minimum inhibitory concentrations (MIC) were determined using the broth micro-dilution method according to the Clinical and Laboratory Standards Institute (CLSI) 2015 guideline. The commercial antimicrobial agents Penicillin and Vancomycin were used as positive controls. After 16–20 h incubation at 37 °C, MIC values were defined as the lowest concentrations of antibiotics with no visible growth of bacteria. MIC values of more than 128 μg/mL were considered as no activity [27]. 

## 4. Conclusions

In conclusion, four new farnesylcyclohexenones (**1**–**4**) and a known analog (**5**) were isolated from a fraction of the EtOAc extract of the deep sea-derived fungus *Penicillium* sp. YPGA11. Peniginsengin A−E (**1**–**5**), characterized by a highly oxygenated 1-methylcyclohexene unit and a rare (4*E*,8*E*)-4,8-dimethyldeca-4,8-dienoic acid side chain, are rarely found in nature. The absolute configurations of peniginsengins were resolved for the first time by ECD calculations in the current study. Compounds **2**–**4** exhibited moderate antibacterial activity against *Staphylococcus aureus*. Our study enriches the structure diversity of deep sea-derived natural products. 

## Figures and Tables

**Table 1:** ^1^H and ^13^C nuclear magnetic resonance (NMR) data of **1** and **2** (**1** in DMSO-*d*_6_ and **2** in CD_3_OD, δ in ppm).

No.	1		2	
	δ_H_^a^ (Mult, *J* in Hz)	δ_C_^b^, Type	δ_H_^c^ (Mult, *J* in Hz)	δ_C_^d^, Type
1		199.6, C		201.4, C
2		77.1, C	2.43, ddd (10.2, 3.8, 2.3)	53.4, CH
3	2.87, d (15.9) 2.80, d (15.9)	49.5, CH_2_	4.20, dd (3.0, 2.3)	73.7, CH
4		197.1, C	4.44, br s	73.3, CH
5		150.3, C		162.4, C
6	6.69, d (1.34)	135.7, CH	5.84, br s	126.8, CH
7	1.89, d (1.34)	15.5, CH_3_	2.02, br s	20.3, CH_3_
1′	2.35, dd (14.0, 7.4) 2.22, dd (14.0, 7.4)	36.8, CH_2_	2.25, m 2.60, ddd (14.5, 6.9, 3.8)	24.4, CH_2_
2′	5.02, t (7.4)	118.4, CH	5.17, t (6.9)	123.2, CH
3′		138.1, C		138.1, C
4′	1.93, m	39.5, CH_2_	2.05, m	40.7, CH_2_
5′	1.99, m	25.8, CH_2_	2.13, m	27.5, CH_2_
6′	5.06, t (6.60)	123.9, CH	5.16, t (5.9)	125.9, CH
7′		133.7 C		134.7, C
8′	2.15, m	34.2, CH_2_	2.27, m	35.7, CH_2_
9′	2.24, m	32.7, CH_2_	2.38, m	34.0, CH_2_
10′		174.2, C		177.4, C
11′	1.55, s	15.8, CH_3_	1.63, s	16.1, CH_3_
12′	1.48, s	16.0, CH_3_	1.66, s	16.2, CH_3_

^a^ Recorded at 400 MHz, ^b^ Recorded at 100 MHz, ^c^ Recorded at 600 MHz, ^d^ Recorded at 150 MHz.

**Table 2:** ^1^H and ^13^C NMR data of **3** and **4** (CD_3_OD, δ in ppm).

No.	3		4	
	δ_H_^a^ (Mult, *J* in Hz)	δ_C_^b^, Type	δ_H_^a^ (Mult, *J* in Hz)	δ_C_^b^, Type
1	4.18, m	67.2, CH		194.9, C
2		63.8, C		61.3, C
3	3.31, m	61.4, CH	3.70, d (2.6)	57.5, CH
4	4.19, m	68.3, CH	5.85, d (2.6)	70.3, CH
5		134.9, C		154.5, C
6	5.23, m	124.9, CH	5.83, br s	125.4, CH
7	1.75, s	19.2, CH_3_	1.89, br s	19.8, CH_3_
1′	2.90, d (14.6, 8.9) 2.12, d (14.6, 8.9)	32.1, CH_2_	2.74, dd (15.2, 7.9) 2.44, dd (15.2, 6.8)	27.0, CH_2_
2′	5.14, m	119.6, CH	5.03, dd (7.9, 6.8)	118.0, CH
3′		139.8 C		140.4, C
4′	2.06, m	40.7, CH_2_	2.02, m	40.6, CH_2_
5′	2.13, m	27.2, CH_2_	2.09, m	27.2, CH_2_
6′	5.13, m	125.7, CH	5.12, dd (7.7, 6.9)	125.6, CH
7′		135.0, C		135.1, C
8′	2.25, t (7.7)	35.9, CH_2_	2.25, m	36.0, CH_2_
9′	2.35, t (7.7)	34.3, CH_2_	2.35, m	34.5, CH_2_
10′		177.7, C		177.8, C
11′	1.67, s	16.4, CH_3_	1.64, s	16.4, CH_3_
12′	1.62, s	16.1, CH_3_	1.62, s	16.1, CH_3_
1-OAc			2.20, s	20.6, CH_3_ 171.9, C

^a 1^H NMR recorded at 600 MHz, ^b 13^C NMR recorded at 150 MHz.

**Table 3:** Antibacterial activity of **1**–**4**.

No.	MIC (μg/mL)	
	*S. aureus* ATCC 25913	*S. aureus* ATCC 43300
**1**	>128	>128
**2**	8	32
**3**	16	>128
**4**	32	64
Penicillin ^a^	<0.5	>128
Vancomycin ^a^	− ^b^	<0.5

^a^ Positive control. ^b^ Not tested

## Supplementary Materials

The following are available online at http://www.mdpi.com/1660-3397/16/10/358/s1, Figures S1–S28: HRESIMS, ^1^H, ^13^C NMR, HSQC, HMBC, NOESY, and ^1^H-^1^H COSY spectra of the new compounds 1–4, Table S1: NMR data of 3 in DMSO-*d*_6_.
