# Supplementary material for: Peniginsengins B–E, New Farnesylcyclohexenones from the Deep Sea-Derived Fungus Penicillium sp. YPGA11
Source: Mar Drugs. 2018 Oct 1;16(10):358. doi: 10.3390/md16100358 (PMC6213461; doi:10.3390/md16100358)
Supplement: Supplementary file 1 [file marinedrugs-16-00358-s001.pdf]

Peniginsengins B–E, New Farnesylcyclohexenones from the Deep Sea-Derived Fungus *Penicillium* sp. YPGA11

Zhongbin Cheng [1,4,†], Wei Xu [2,†], Lijun Liu [1], Shumin Li [1], Wangjun Yuan [1,3], Zhuhua Luo [2], Jingjie Zhang [1], Yongjun Cheng [1], and Qin Li [1,3,*]

[1] College of Pharmacy, Henan University, Kaifeng 475004, China; czb360@126.com (Z.C.); 15736871748@163.com (L.L.); lishumin417@163.com (S.L.); yuanwangjun@henu.edu.cn (W.Y.); zhang521571@163.com (J.Z.); chyj15926@163.com (Y.C.)

[2] Key Laboratory of Marine Biogenetic Resources, Third Institute of Oceanography, State Oceanic Administration, Xiamen 361005, China; xuwei@tio.org.cn (W.X.); luozhuhua@tio.org.cn (Z.L.)

[3] Eucommia Ulmoides Cultivation and Utilization of Henan Engineering Laboratory, Kaifeng 475004, China

[4] College of Chemistry and Chemical Engineering, Henan University, Kaifeng 475004, China

* Correspondence: liqin6006@163.com (Q.L.); Tel.: +86-371-2388-3849

† These authors contributed equally to this work.

Abstract: Chemical examination of the EtOAc extract of the deep sea-derived fungus *Penicillium* sp. YPGA11 resulted in the isolation of four new farnesylcyclohexenones, peniginsengins B–E (**1**–**4**), and a known analog peniginsengin A (**5**). The structures of compounds **1**–**4** were determined on the basis of comprehensive analyses of the nuclear magnetic resonance (NMR) and mass spectroscopy (MS) data, and the absolute configurations of **1**, **2**, and **4** were determined by comparisons of experimental electronic circular dichroism (ECD) with calculated ECD spectra. Compounds **1**–**5**, characterized by a highly oxygenated 1-methylcyclohexene unit and a (4*E*,8*E*)-4,8-dimethyldeca-4,8-dienoic acid side chain, are rarely found in nature. Compounds **2**–**4** exhibited antibacterial activity against *Staphylococcus aureus*.

Keywords: *Penicillium* sp.; deep sea-derived fungus; farnesylcyclohexenones; antibacterial

Content

Figure S1 ^{1}H NMR Spectrum of **1** in DMSO-d_6 (400 MHz)................................1

Figure S2 ^{13}C NMR Spectrum of **1** in DMSO-d_6 (100 MHz)...............................1

Figure S3 HSQC Spectrum of **1** in DMSO-d_6 (400 MHz) 2

Figure S4 ^{1}H-^{1}H COSY Spectrum of **1** in DMSO-d_6 (400 MHz)..........................2

Figure S5 HMBC Spectrum of **1** in DMSO-d_6 (400 MHz) 3

Figure S6 NOESY Spectrum of **1** in DMSO-d_6 (400 MHz).................................3

Figure S7 ^{1}H NMR Spectrum of **2** in Methanol-d_4 (600 MHz).............................4

Figure S8 ^{13}C NMR Spectrum of **2** in Methanol-d_4 (150 MHz)............................4

Figure S9 HSQC Spectrum of **2** in Methanol-d_4 (600 MHz)................................5

Figure S10 ^{1}H-^{1}H COSY Spectrum of **2** in Methanol-d_4 (600 MHz)......................5

Figure S11 HMBC Spectrum of **2** in Methanol-d_4 (600 MHz)...............................6

Figure S12 NOESY Spectrum of **2** in Methanol-d_4 (600 MHz) 6

Figure S13 ^{1}H NMR Spectrum of **3** in Methanol-d_4 (600 MHz).............................7

Figure S14 ^{13}C NMR Spectrum of **3** in Methanol-d_4 (150 MHz)............................7

Figure S15 HSQC Spectrum of **3** in Methanol-d_4 (600 MHz)................................8

Figure S16 ^{1}H-^{1}H COSY Spectrum of **3** in Methanol-d_4 (600 MHz)......................8

Figure S17 HMBC Spectrum of **3** in Methanol-d_4 (600 MHz)...............................9

Figure S18 NOESY Spectrum of **3** in Methanol-d_4 (600 MHz) 9

Figure S19 ^{1}H NMR Spectrum of **4** in Methanol-d_4 (600 MHz)...........................10

Figure S20 ^{13}C NMR Spectrum of **4** in Methanol-d_4 (150 MHz)..........................10

Figure S21 HSQC Spectrum of **4** in Methanol-d_4 (600 MHz)..............................11

Figure S22 ^1H-^1H COSY Spectrum of **4** in Methanol-d_4 (600 MHz) 11

Figure S23 HMBC Spectrum of **4** in Methanol-d_4 (600 MHz) 12

Figure S24 NOESY Spectrum of **4** in Methanol-d_4 (600 MHz) 12

Figure S25 HRESIMS spectra of **1** 13

Figure S26 HRESIMS spectra of **2** 13

Figure S27 HRESIMS spectra of **3** 14

Figure S28 HRESIMS spectra of **4** 14

Tables S1 NMR data of **3** in DMSO-d_6 15

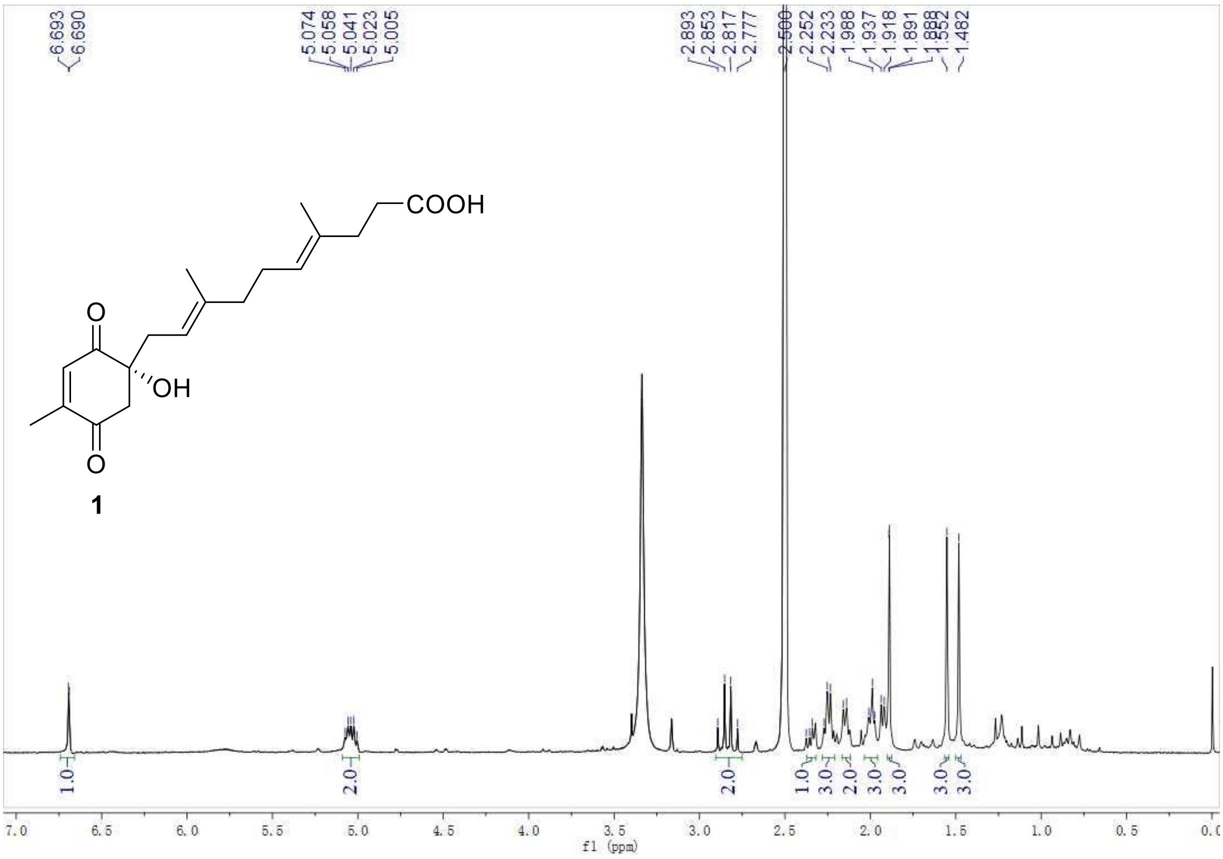

Figure S1. ^{1}H NMR Spectrum of **1** in DMSO-d_6 (400 MHz).

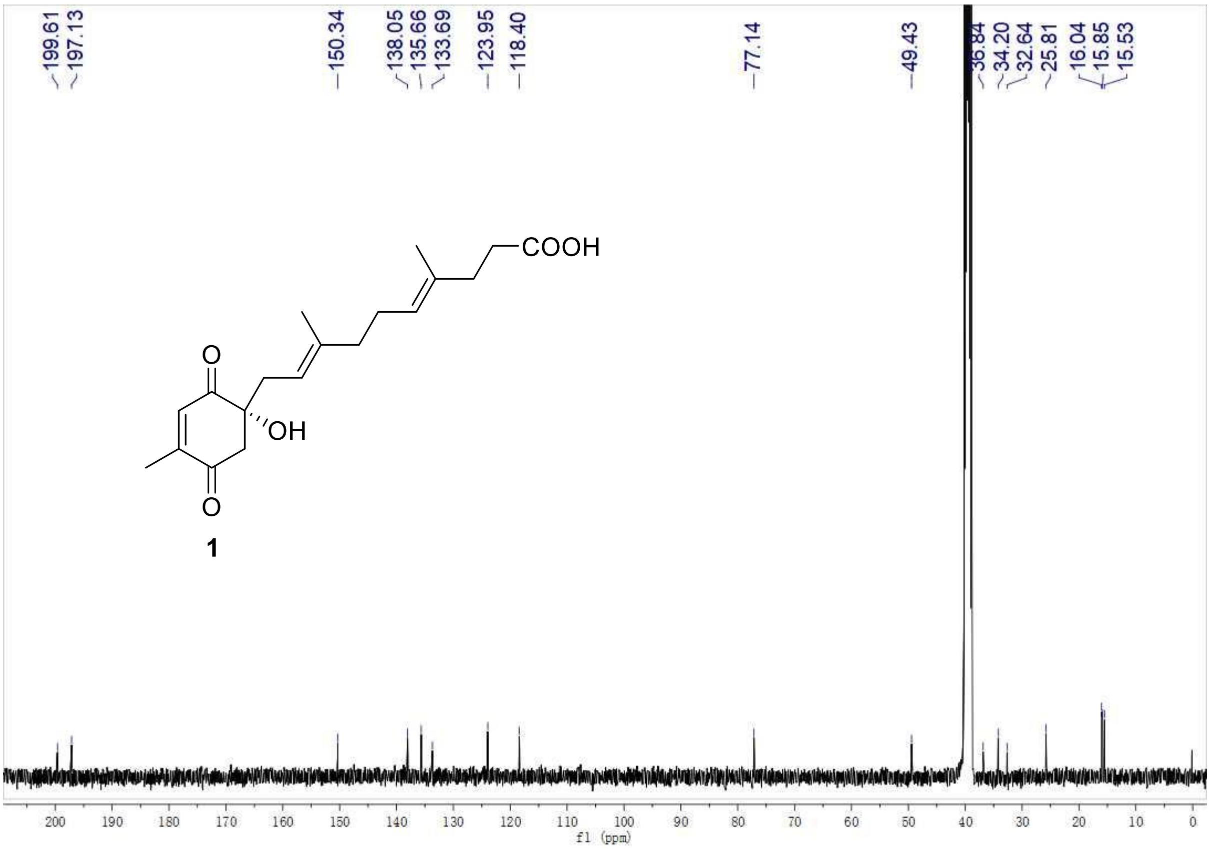

Figure S2. ^{13}C NMR Spectrum of **1** in DMSO-d_6 (100 MHz).

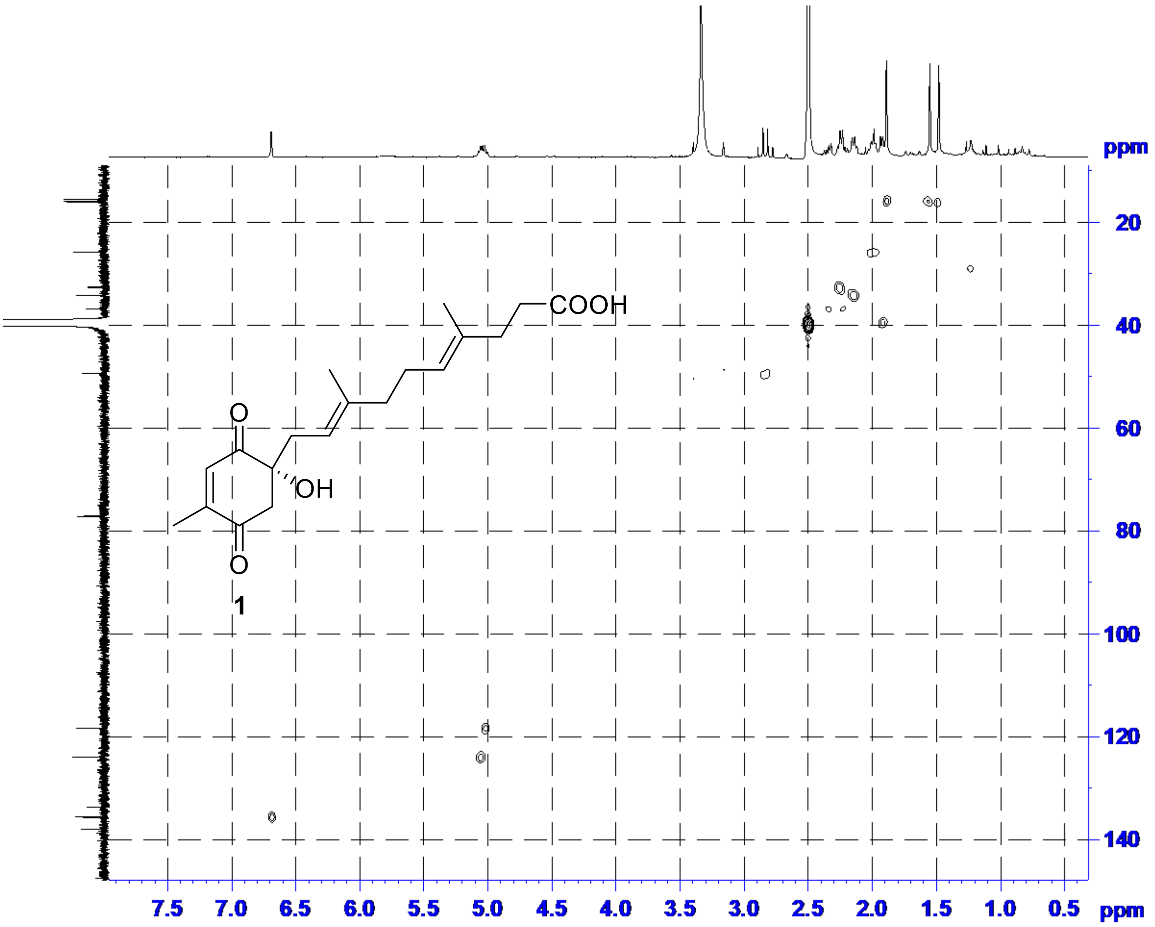

Figure S3. HSQC Spectrum of **1** in DMSO-d_6 (400 MHz)

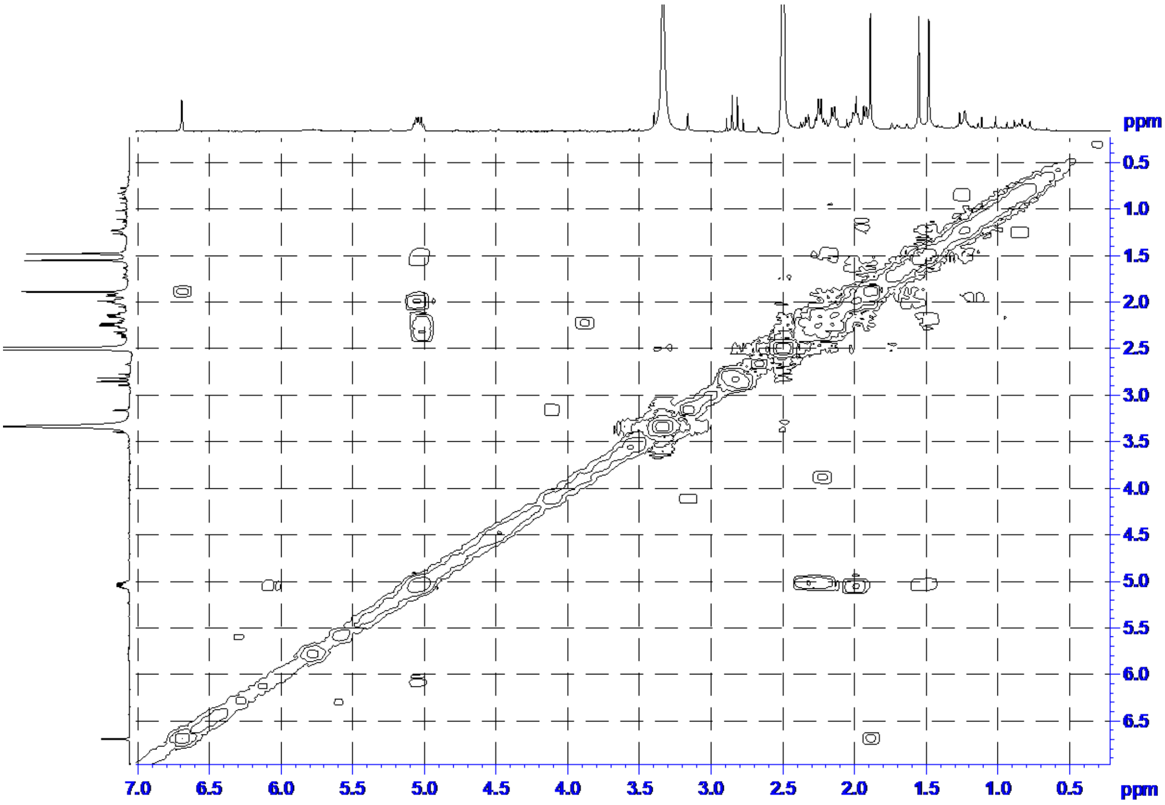

Figure S4. COSY Spectrum of **1** in DMSO-d_6 (400 MHz)

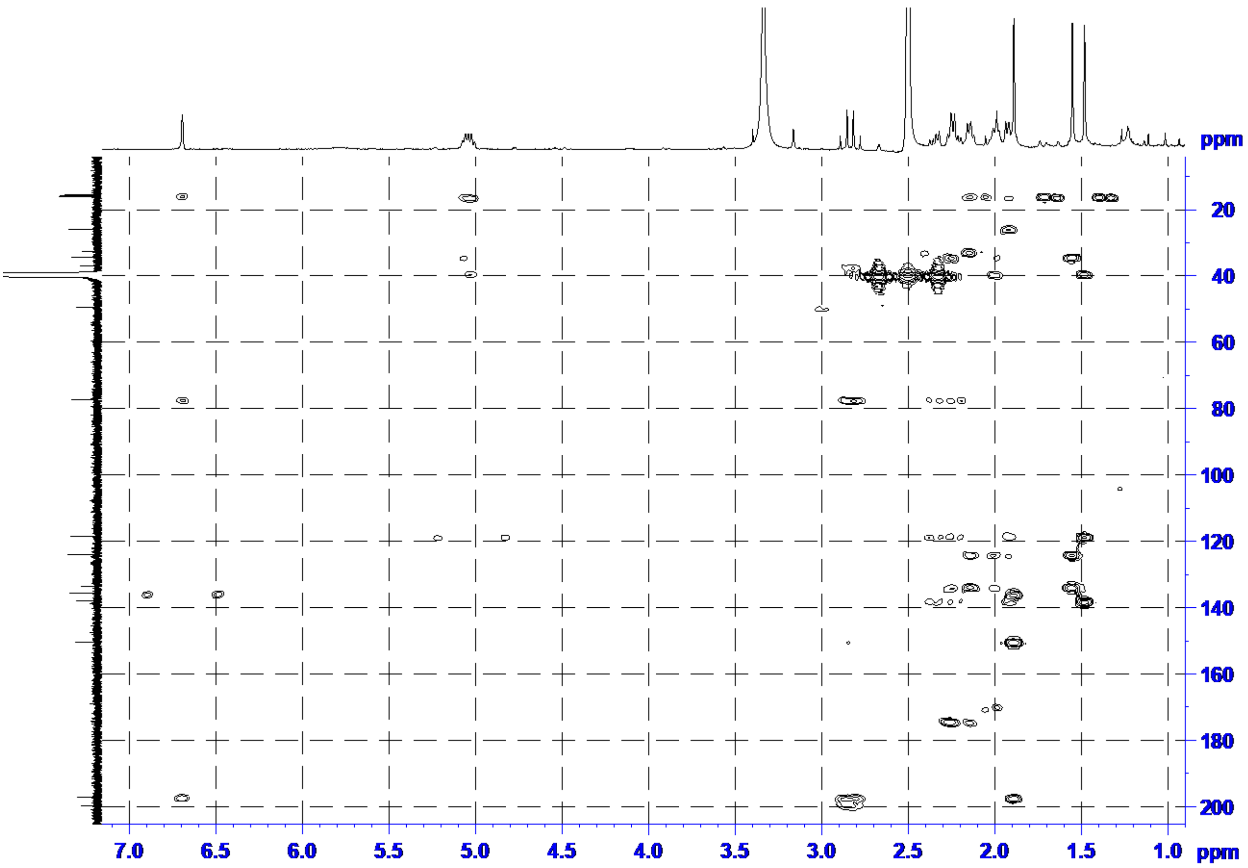

Figure S5. HMBC Spectrum of **1** in DMSO-d_6 (400 MHz)

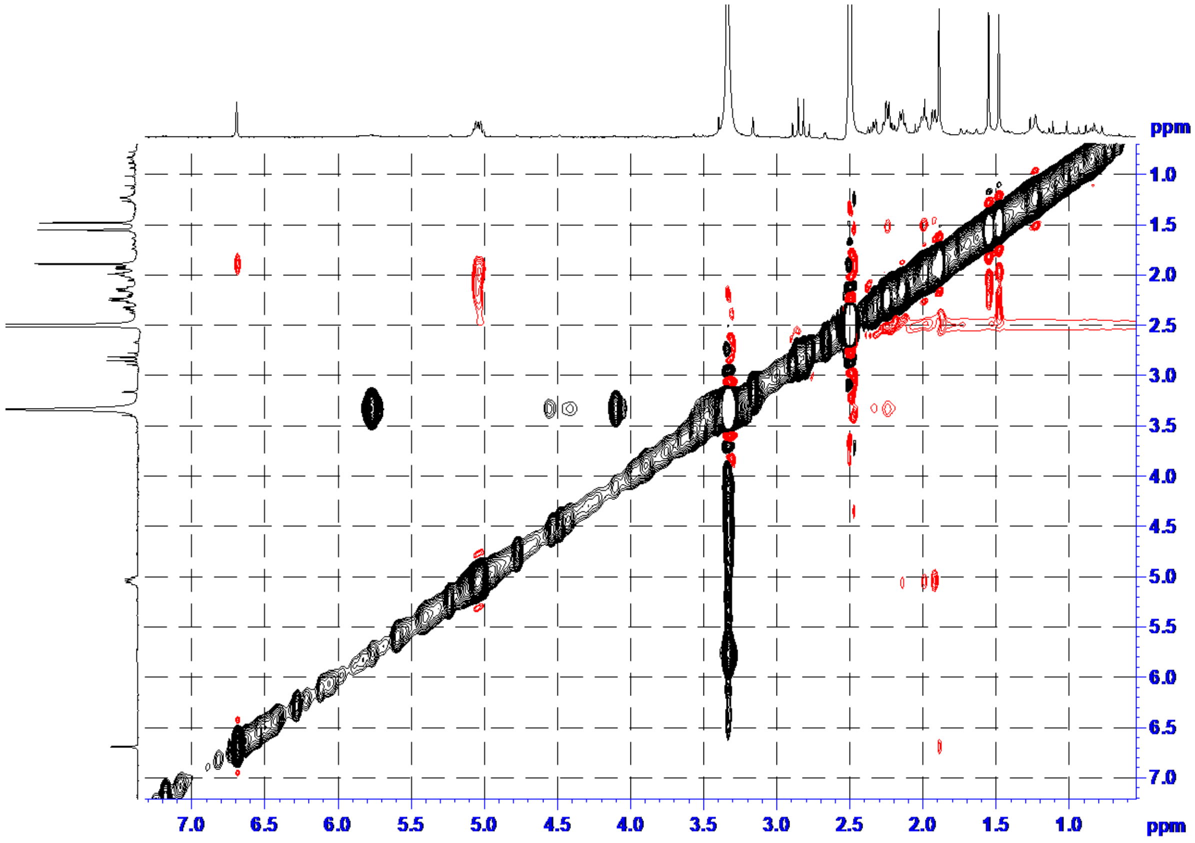

Figure S6. NOESY Spectrum of **1** in DMSO-d_6 (400 MHz)

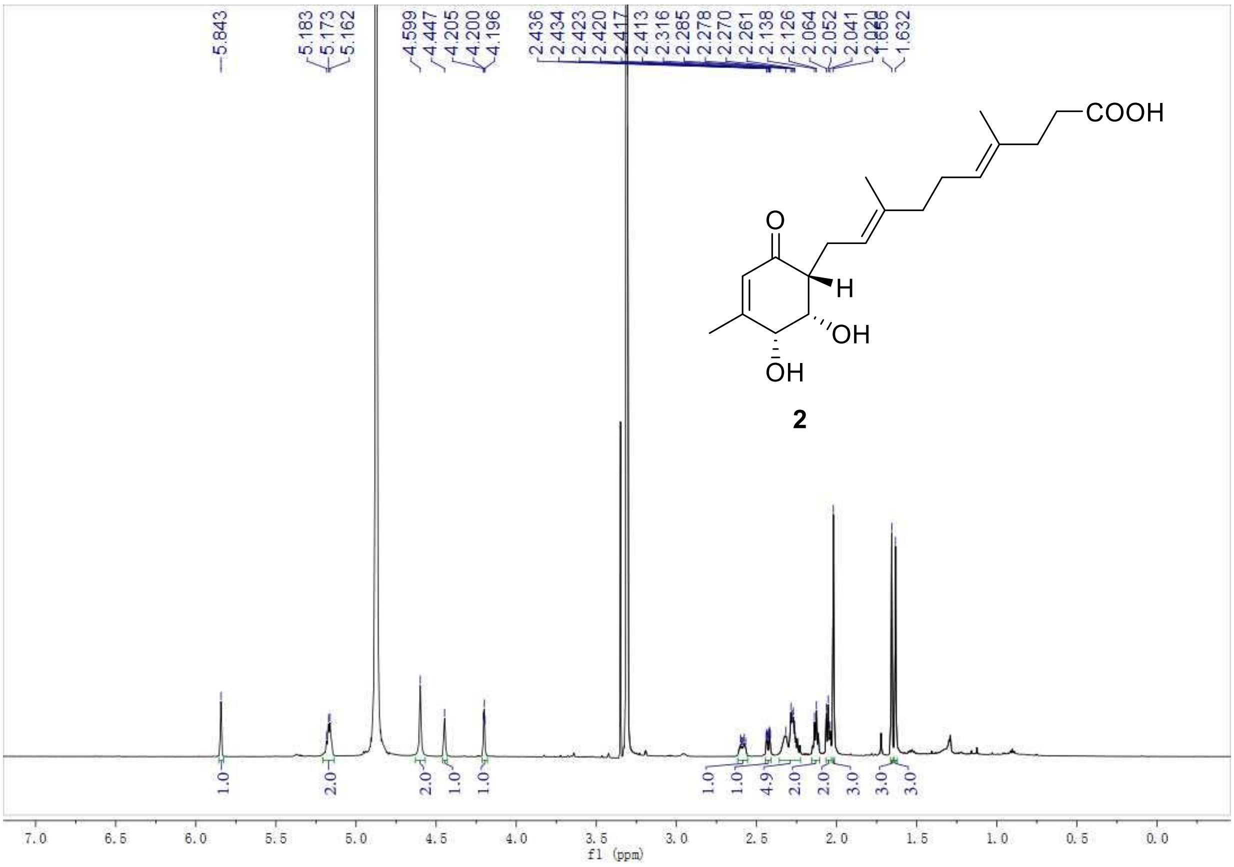

Figure S7. ^{1}H NMR Spectrum of **2** in Methanol-d_4 (600 MHz).

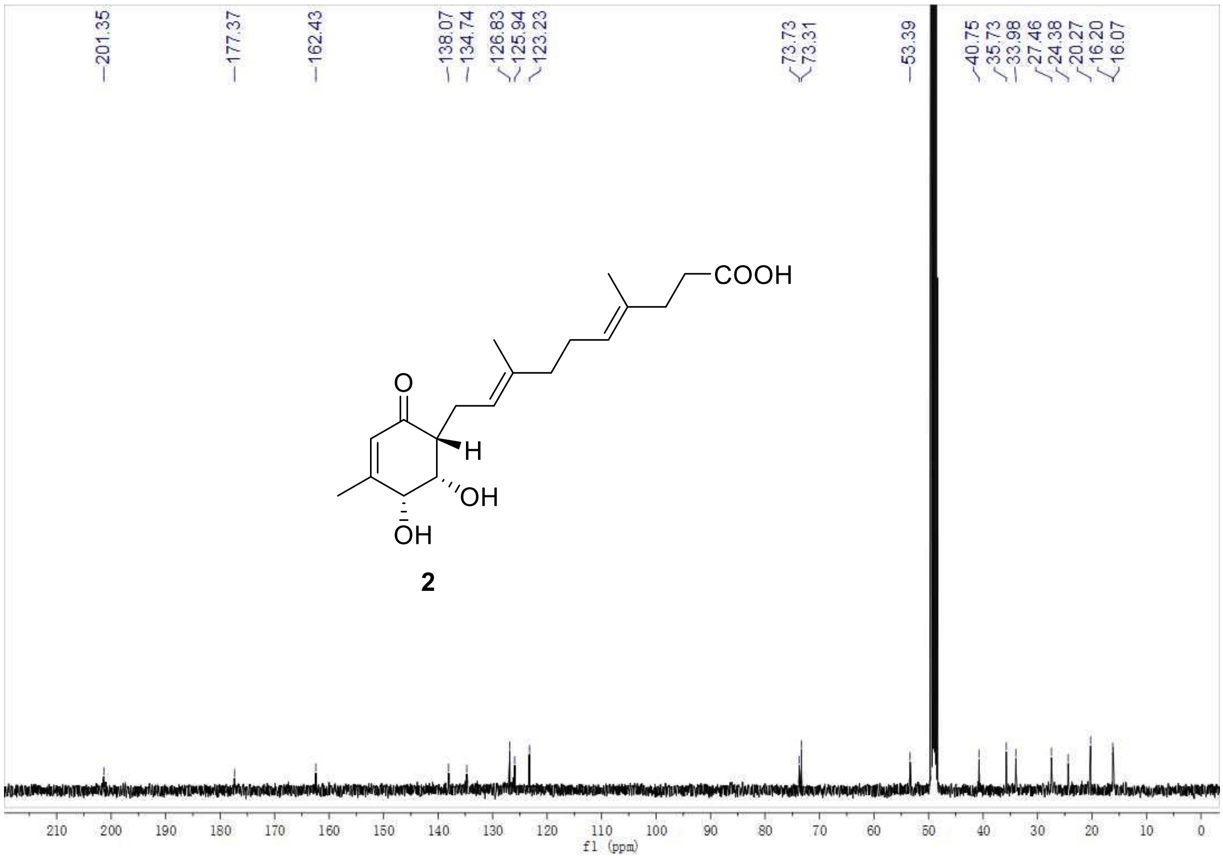

Figure S8. ^{13}C NMR Spectrum of **2** in Methanol-d_4 (150 MHz).

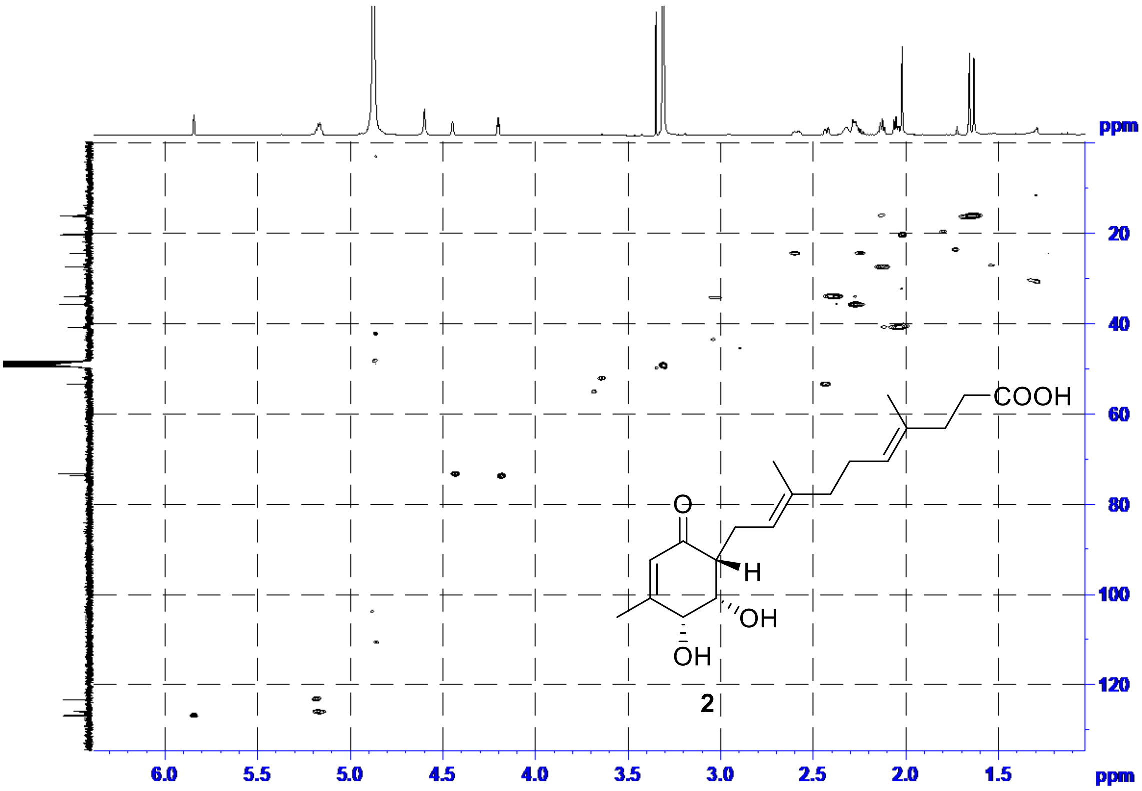

Figure S9. HSQC Spectrum of **2** in Methanol-d_4 (600 MHz).

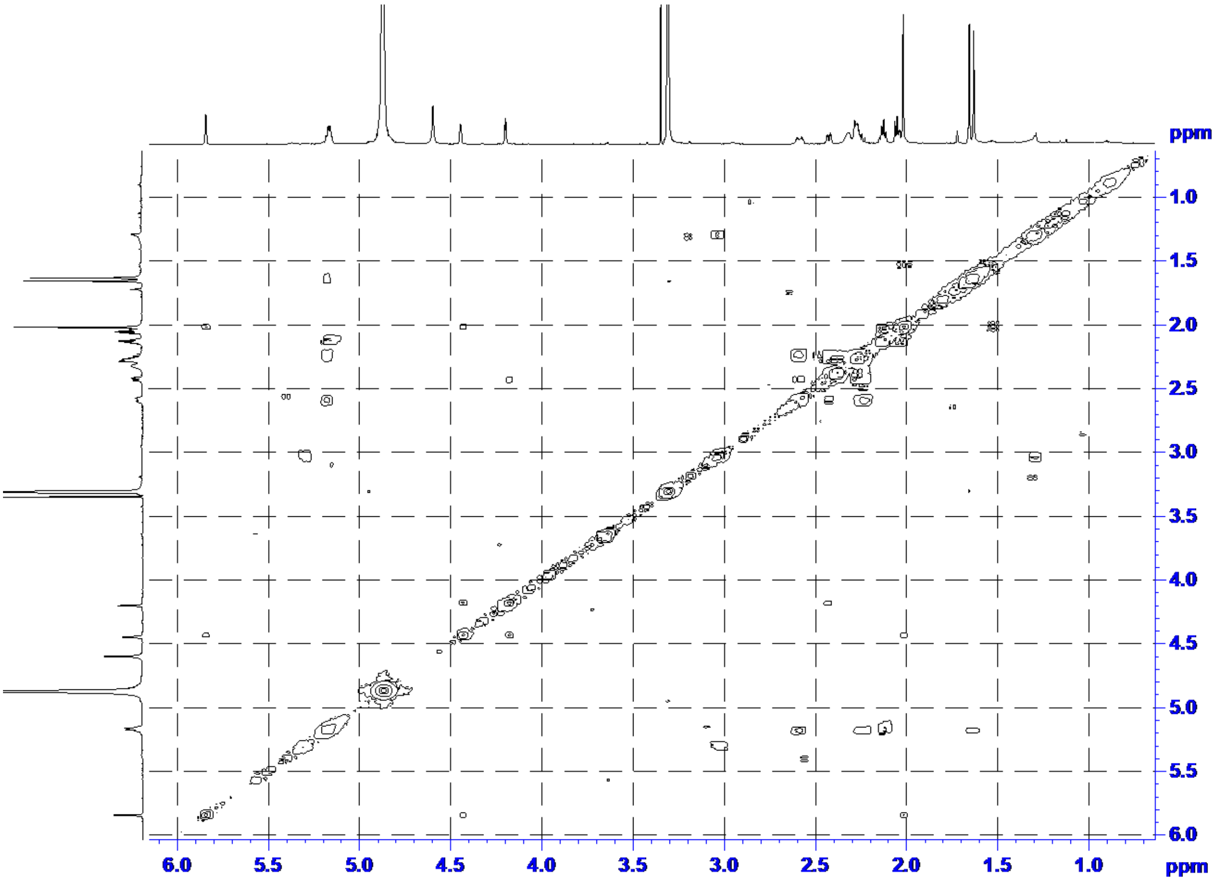

Figure S10. COSY Spectrum of **2** in Methanol-d_4 (600 MHz).

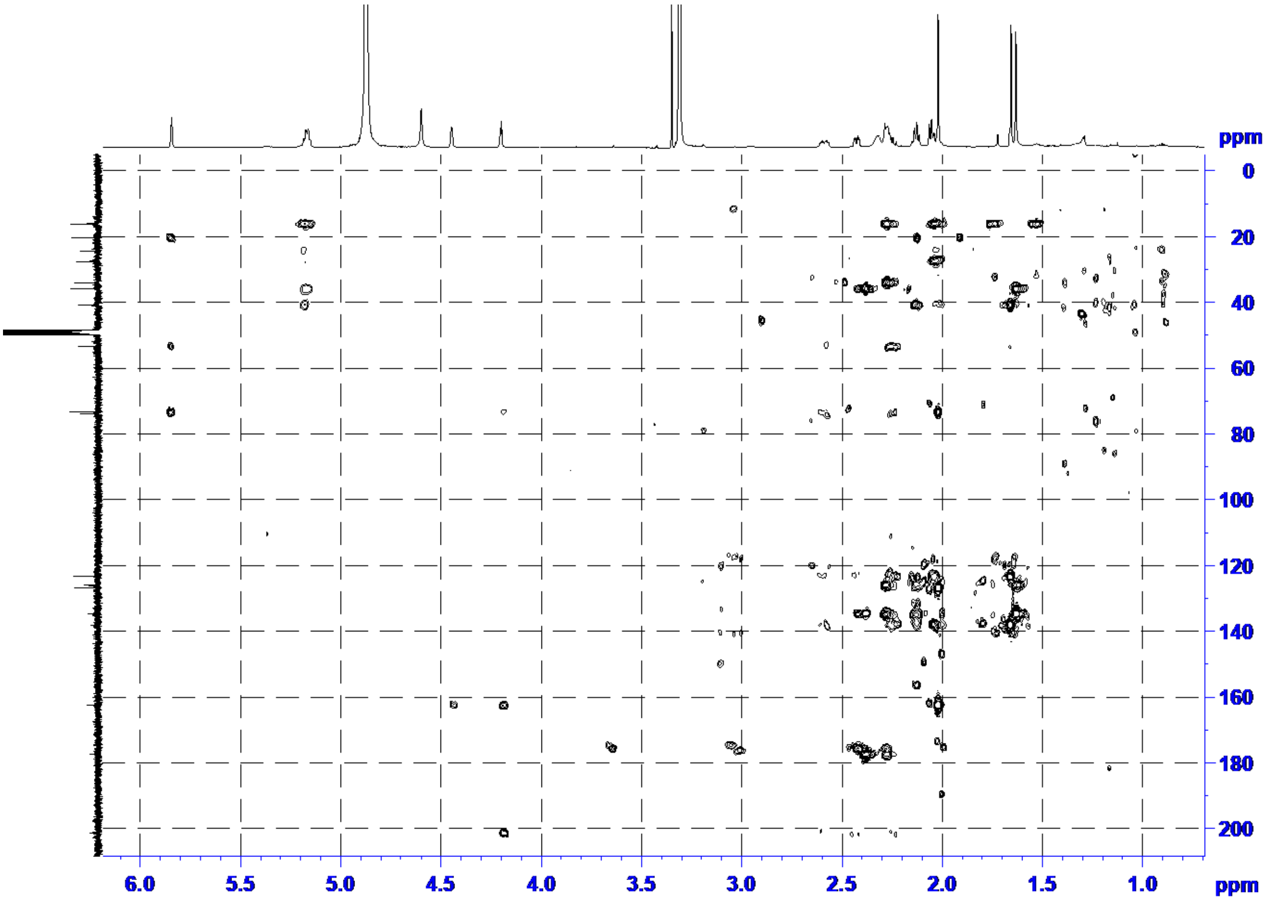

Figure S11. HMBC Spectrum of **2** in Methanol-d_4 (600 MHz).

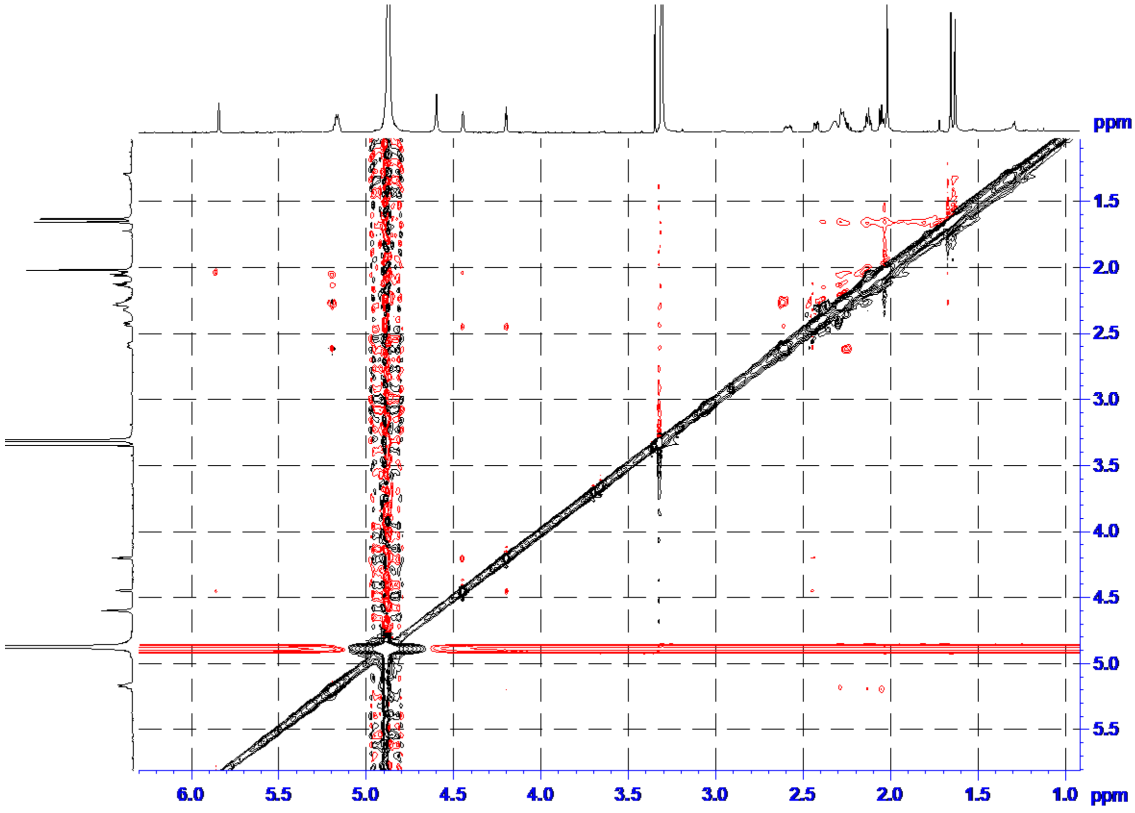

Figure S12. NOESY Spectrum of **2** in Methanol-d_4 (600 MHz).

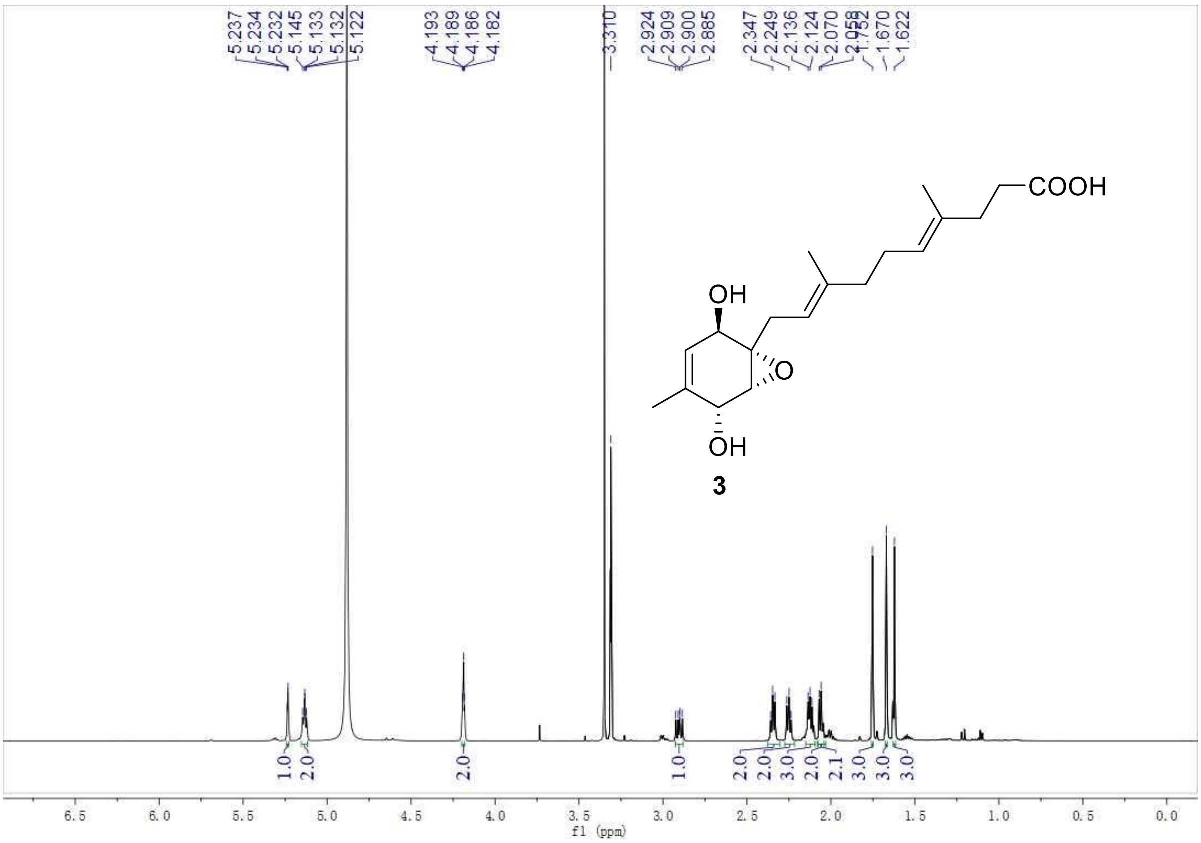

Figure S13. ^{1}H NMR Spectrum of **3** in Methanol-d_4 (600 MHz).

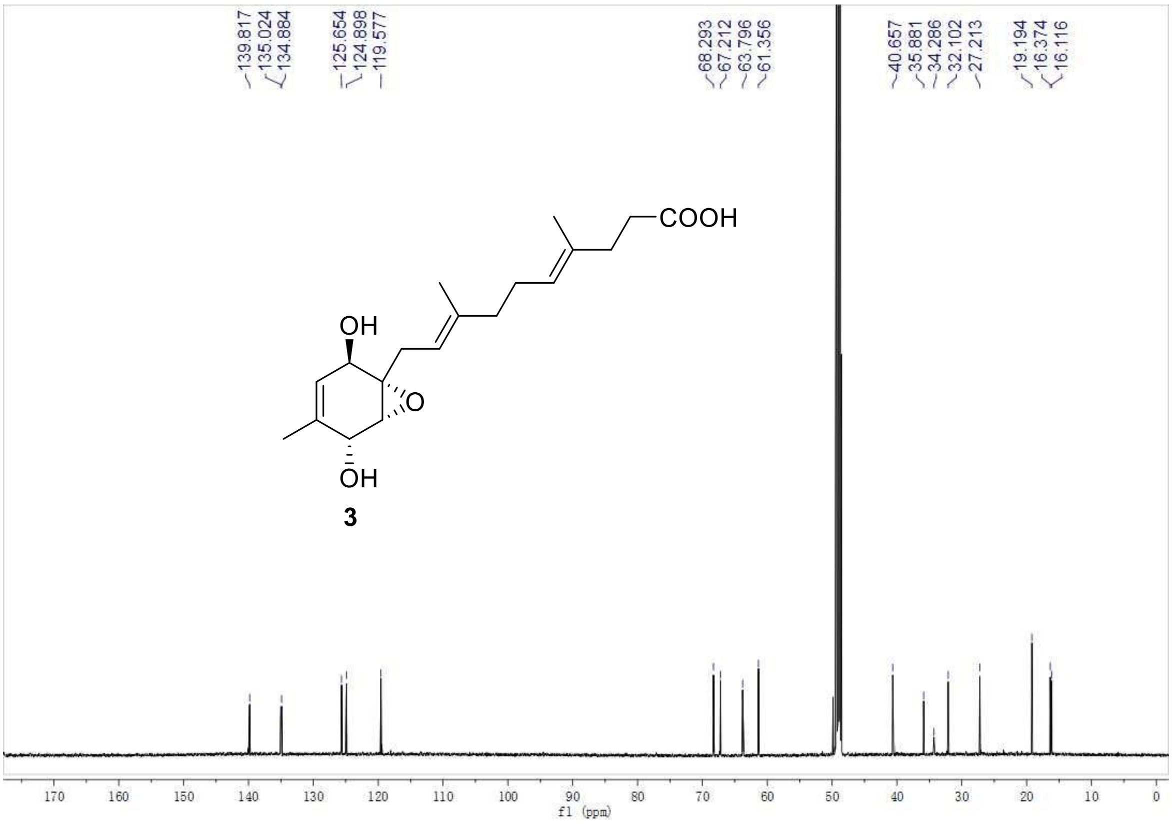

Figure S14. ^{13}C NMR Spectrum of **3** in Methanol-d_4 (150 MHz).

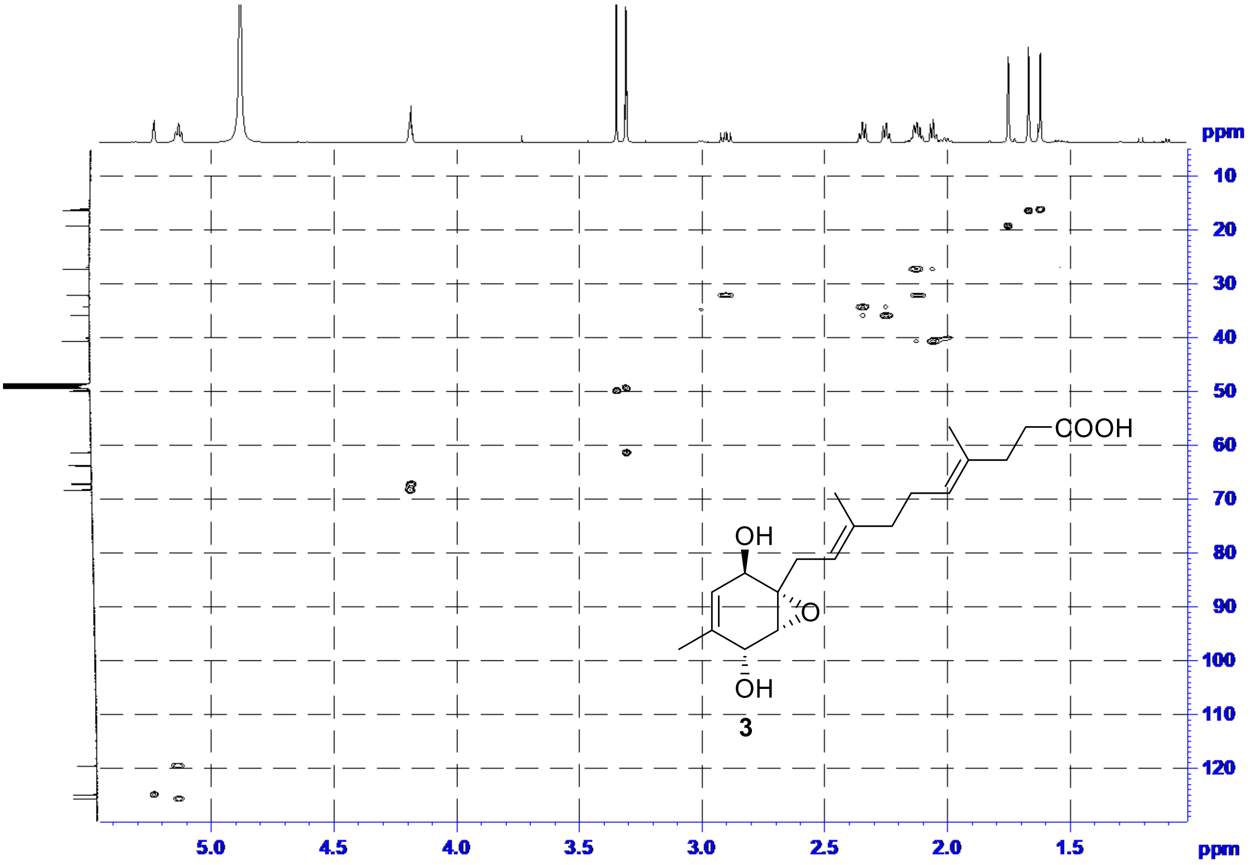

Figure S15. HSQC Spectrum of **3** in Methanol-d_4 (600 MHz).

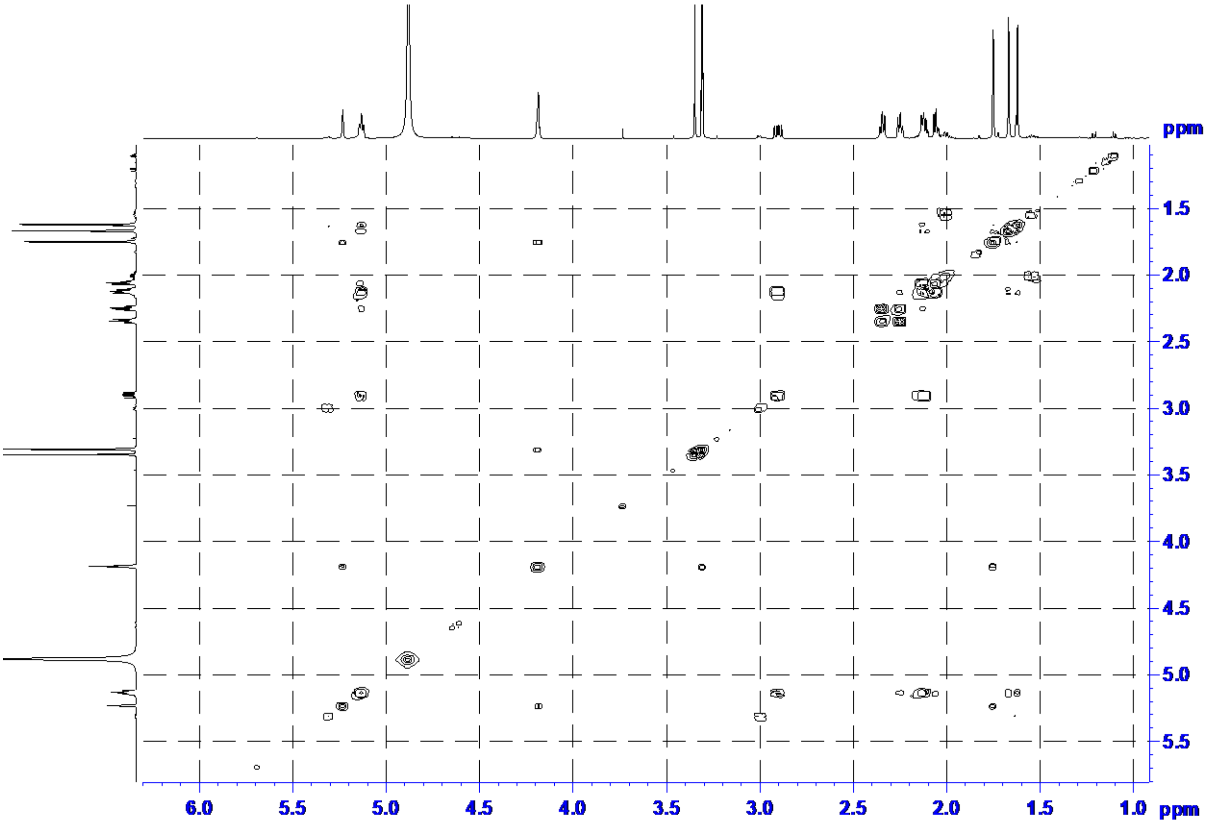

Figure S16. COSY Spectrum of **3** in Methanol-d_4 (600 MHz).

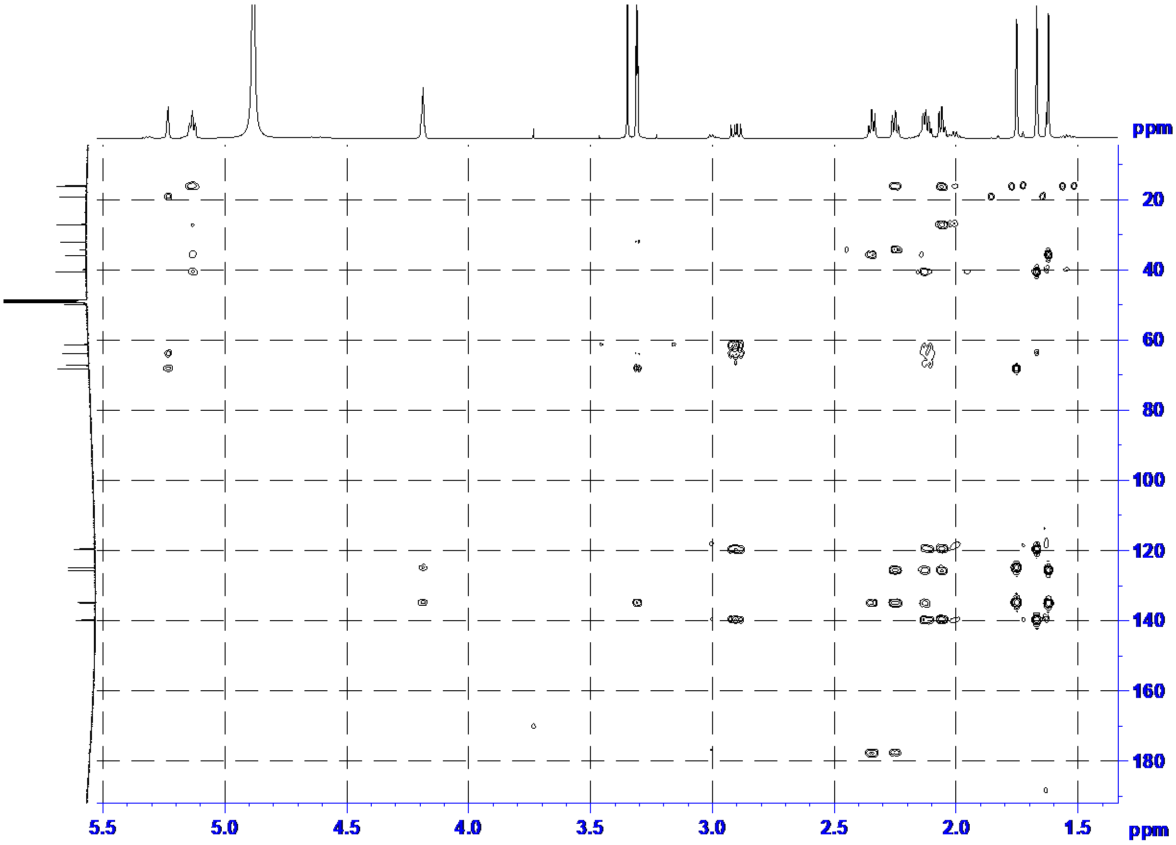

Figure S17. HMBC Spectrum of **3** in Methanol-d_4 (600 MHz).

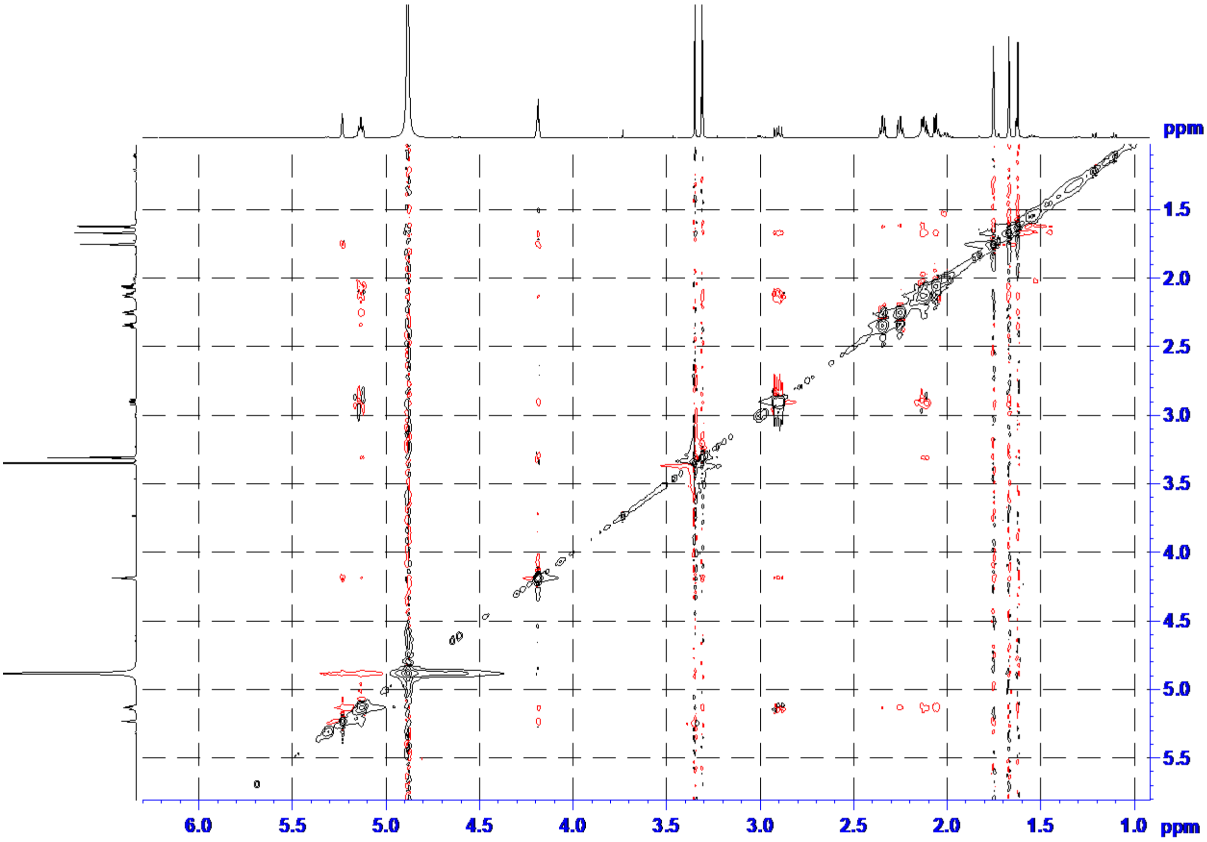

Figure S18. NOESY Spectrum of **3** in Methanol-d_4 (600 MHz).

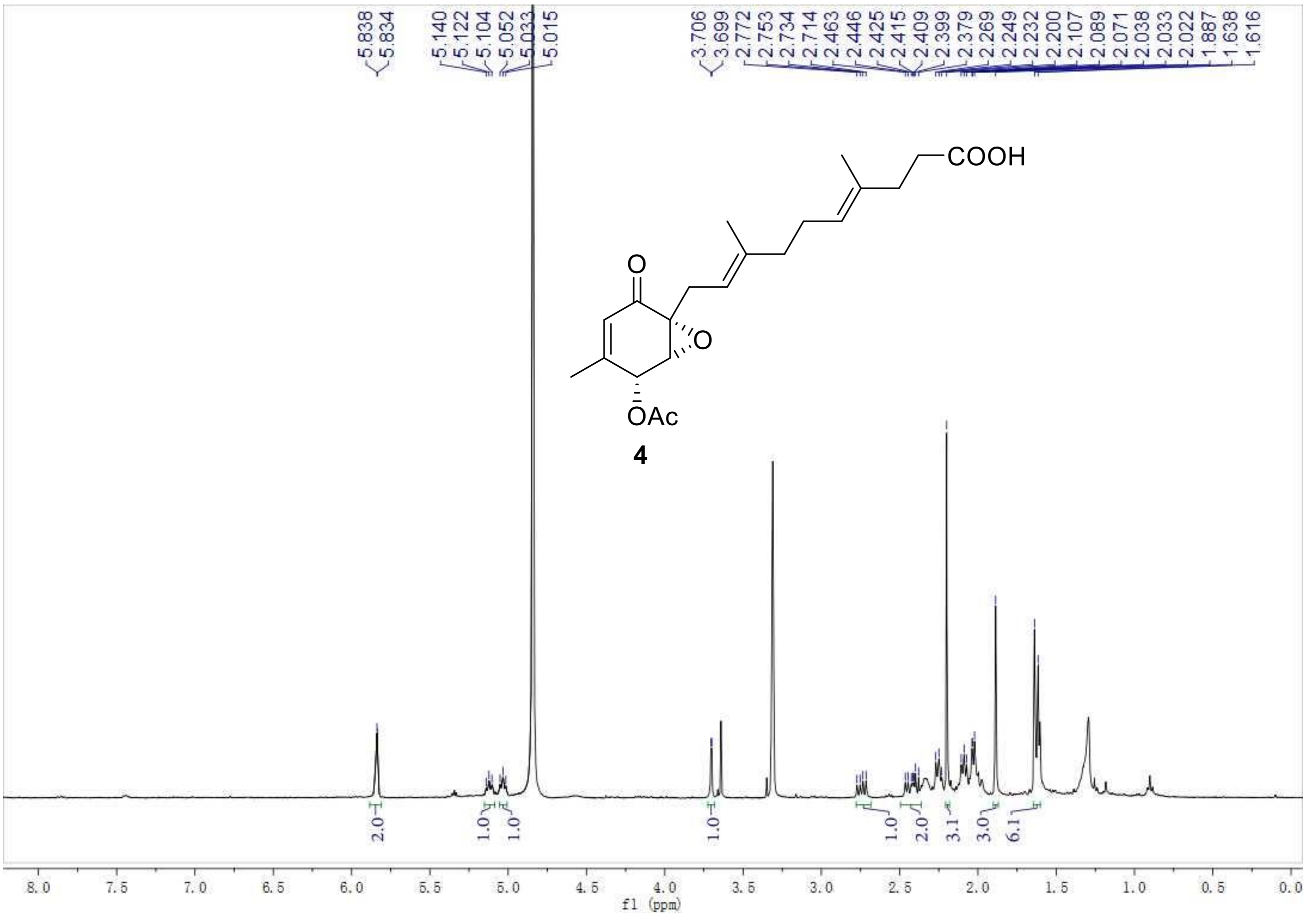

Figure S19. ^{1}H NMR Spectrum of **4** in Methanol-d_4 (600 MHz).

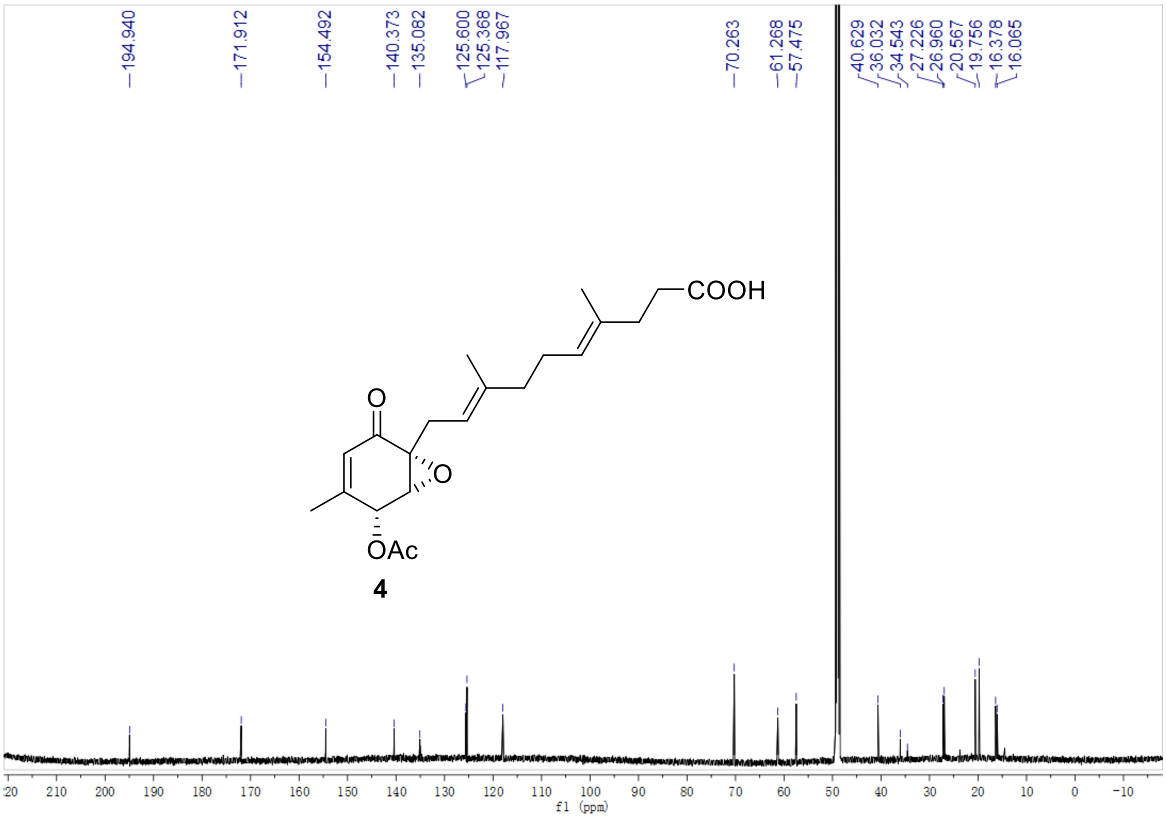

Figure S20. ^{13}C NMR Spectrum of **4** in Methanol-d_4 (150 MHz).

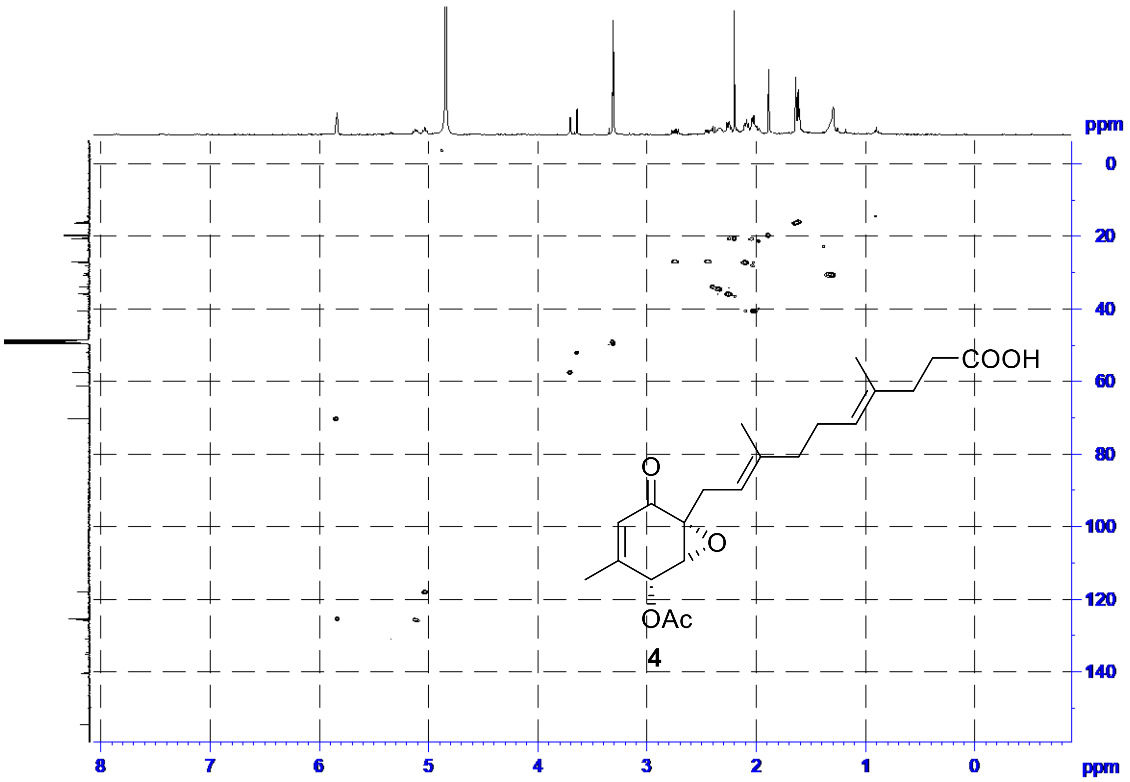

Figure S21. HSQC Spectrum of **4** in Methanol-d_4 (600 MHz).

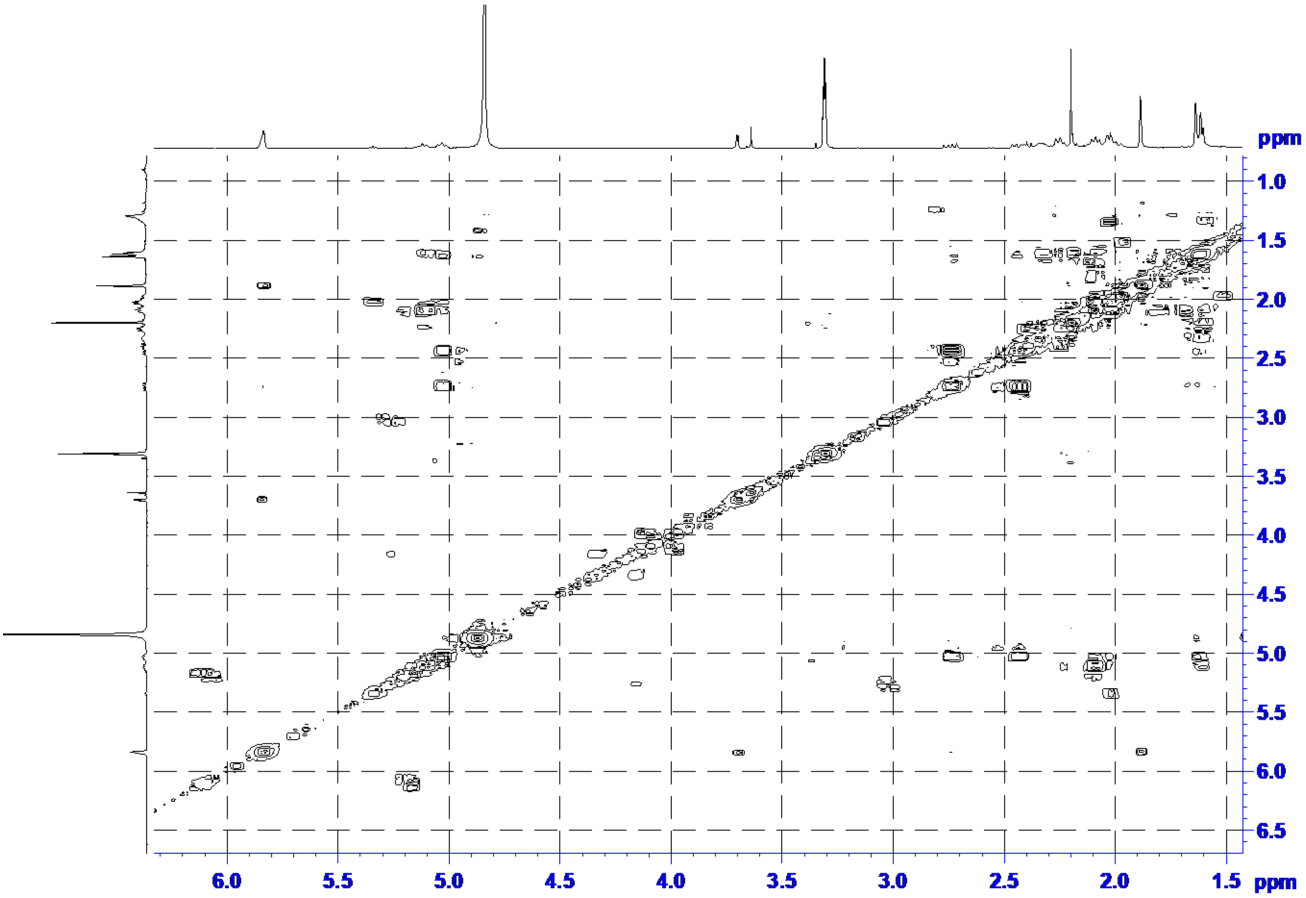

Figure S22. COSY Spectrum of **4** in Methanol-d_4 (600 MHz).

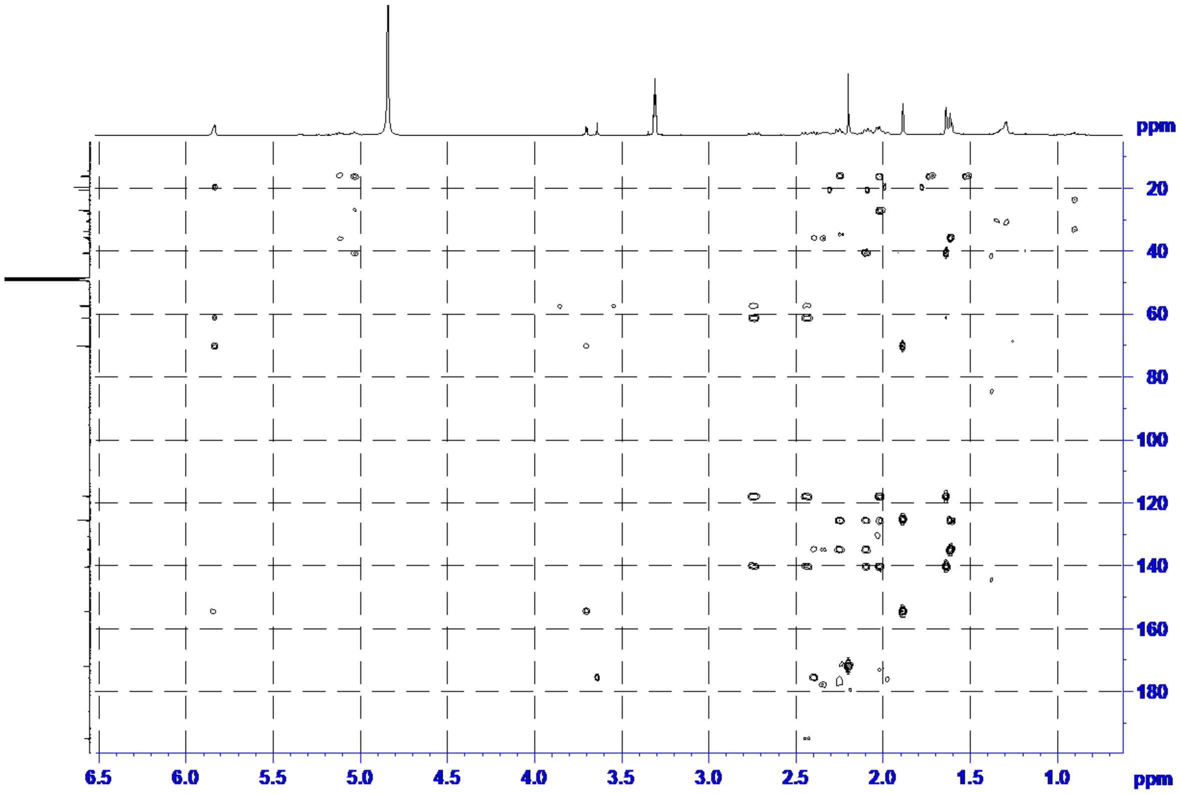

Figure S23. HMBC Spectrum of **4** in Methanol-d_4 (600 MHz).

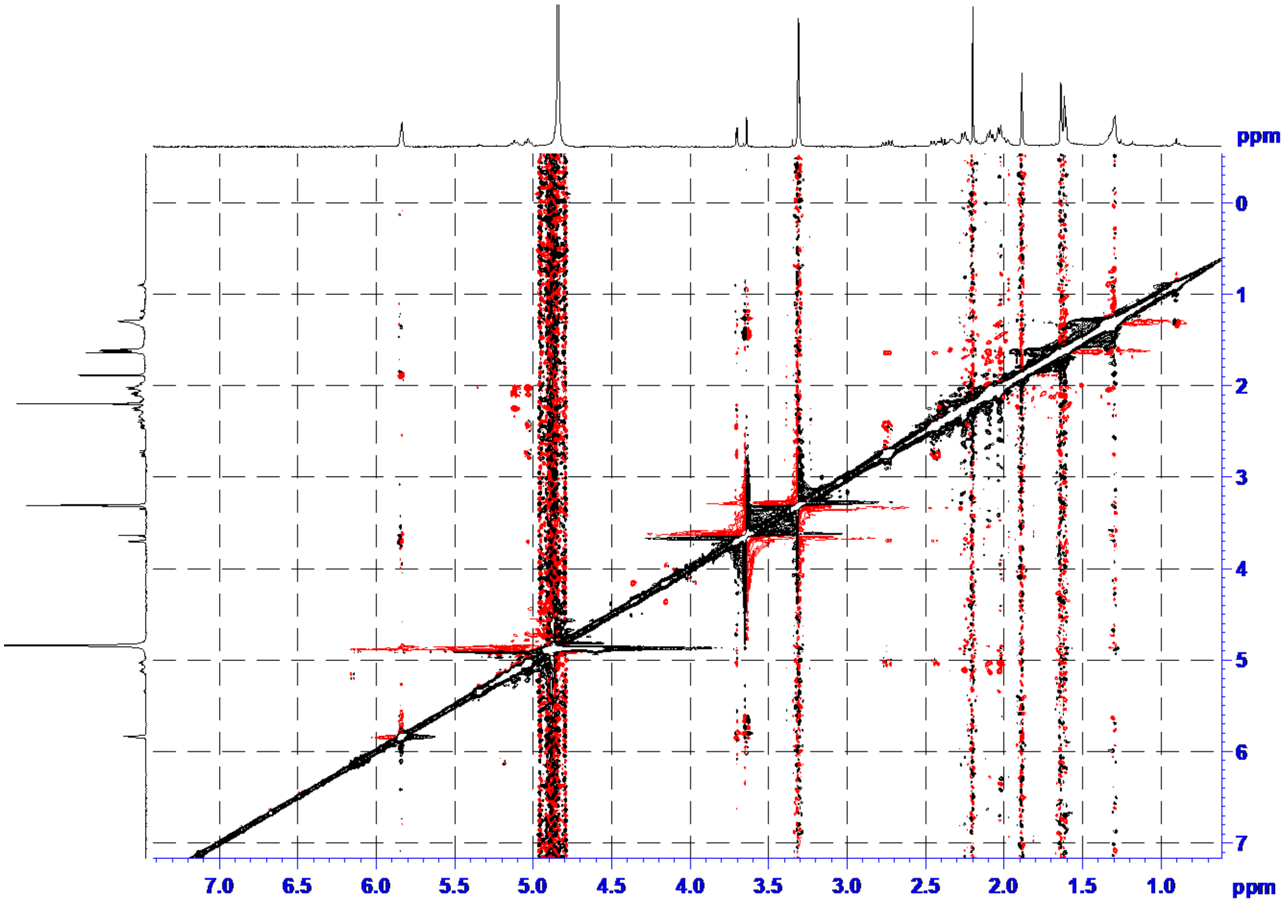

Figure S24. NOESY Spectrum of **4** in Methanol-d_4 (600 MHz).

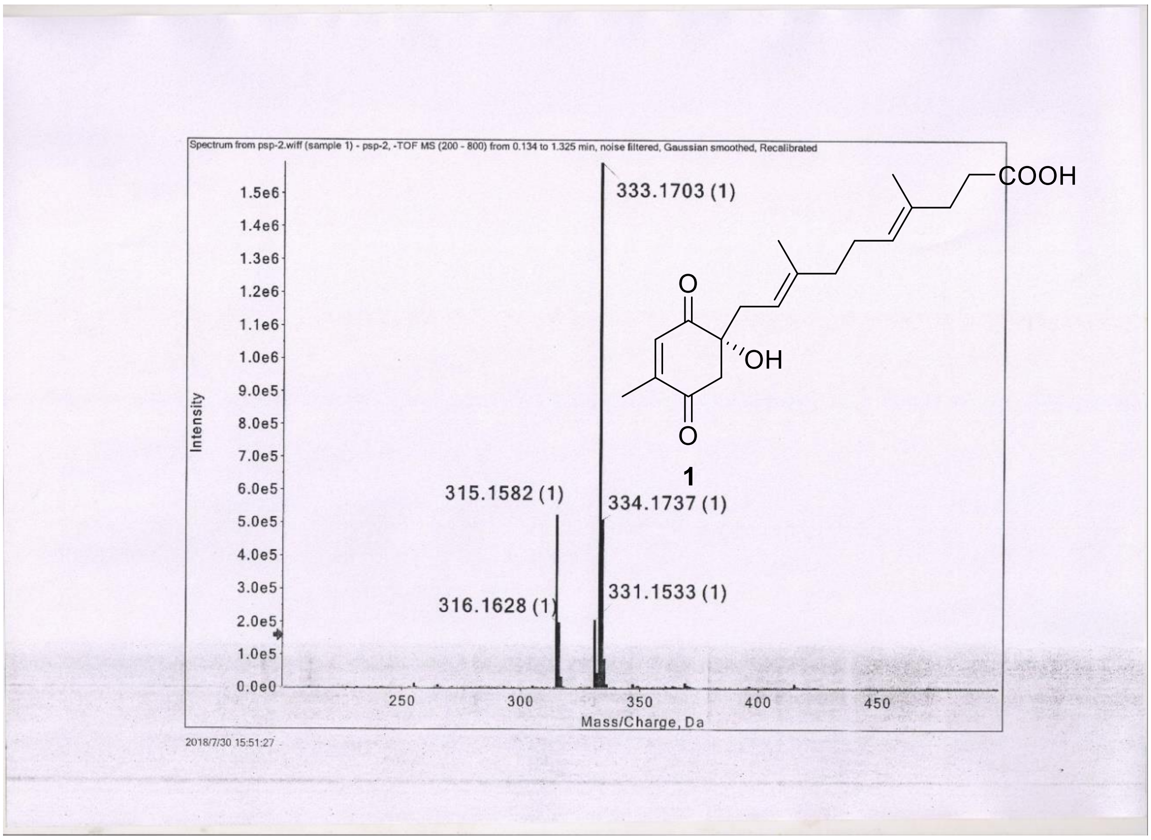

Figure S25. HRESIMS spectrum of **1**

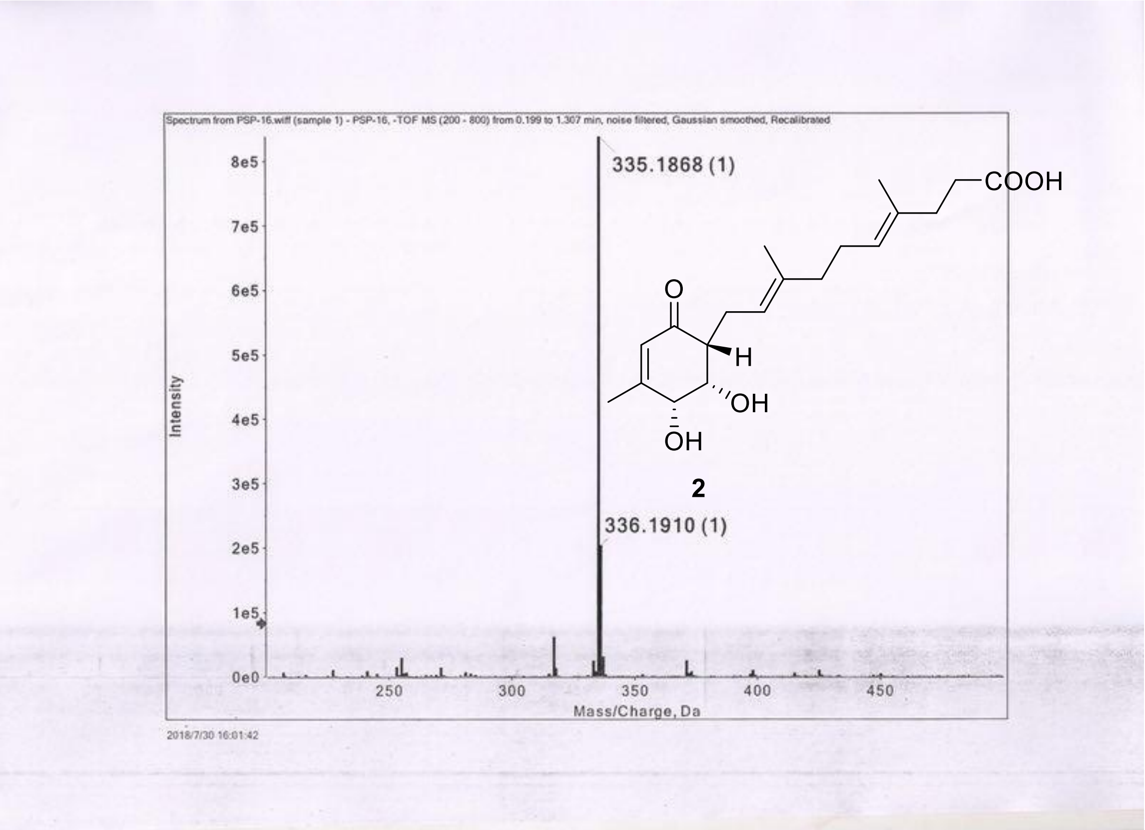

Figure S26. HRESIMS spectrum of **2**

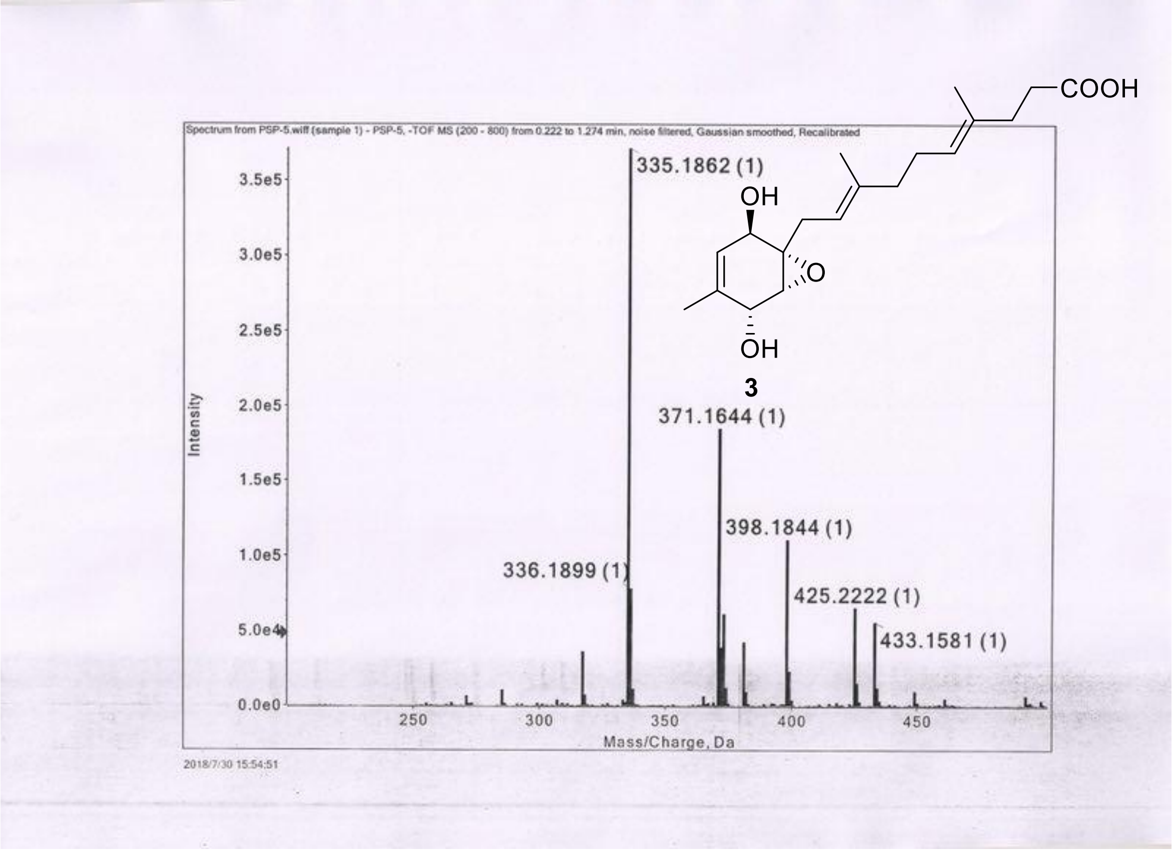

Figure S27. HRESIMS spectrum of **3**

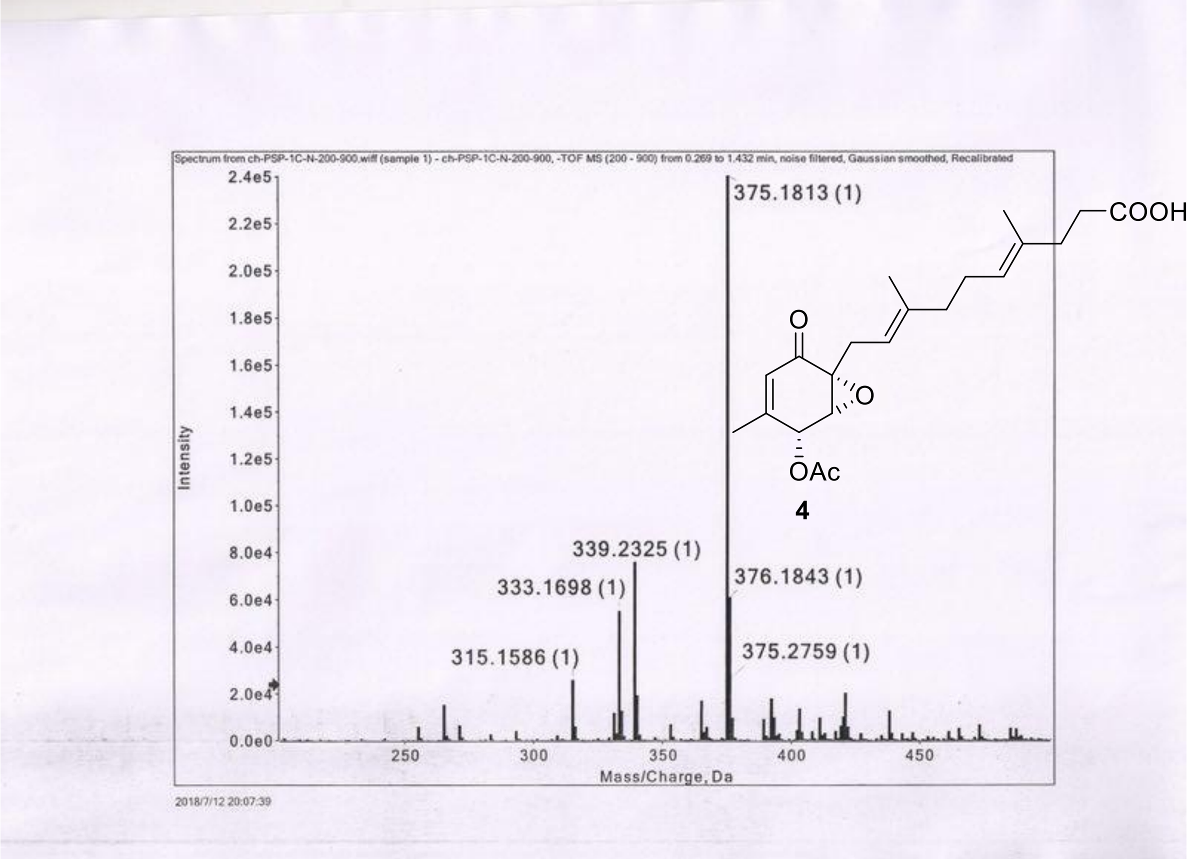

Figure S28. HRESIMS spectrum of **4**

Table S1. ^{1}H and ^{13}C NMR Data of Compounds **3** in DMSO-d_6

	**3**	
NO.	δ_H [a]	δ_C [b]
1	4.05, m	65.2, CH
2		62.0, C
3	3.13, d (2.3)	59.4, CH
4	4.07, d (2.3)	66.2, CH
5		132.8, C
6	5.07, m	123.9, CH
7	1.62, s	15.5, CH_3
1′	2.77, d (14.4, 8.7)	30.7, CH_2
	2.01, d (14.4, 8.7)	
2′	5.07, m	118.8, CH
3′		137.4, C
4′	1.97, m	39.2, CH_2
5′	2.04, m	25.9, CH_2
6′	5.07, m	124.0, CH
7′		133.7, C
8′	2.14, m	34.2, CH_2
9′	2.25, m	32.7, CH_2
10′		174.2, C
11′	1.55, s	15.9, CH_3
12′	1.60, s	16.1, CH_3

[a] Recorded at 400 MHz, [b] Recorded at 100 MHz, chemical shifts are in ppm, coupling constants *J* is in Hz.
